# Super-resolution visualization of distinct stalled and broken replication fork structures

**DOI:** 10.1371/journal.pgen.1009256

**Published:** 2020-12-28

**Authors:** Donna R. Whelan, Wei Ting C. Lee, Frances Marks, Yu Tina Kong, Yandong Yin, Eli Rothenberg

**Affiliations:** 1 Department of Pharmacy and Biomedical Sciences, La Trobe Institute for Molecular Science, La Trobe University, Bendigo, Victoria, Australia; 2 Department of Biochemistry and Molecular Pharmacology, Perlmutter Cancer Center, New York University School of Medicine, New York, New York, United States of America; Harvard Medical School, UNITED STATES

## Abstract

Endogenous genotoxic stress occurs in healthy cells due to competition between DNA replication machinery, and transcription and topographic relaxation processes. This causes replication fork stalling and regression, which can further collapse to form single-ended double strand breaks (seDSBs). Super-resolution microscopy has made it possible to directly observe replication stress and DNA damage inside cells, however new approaches to sample preparation and analysis are required. Here we develop and apply multicolor single molecule microscopy to visualize individual replication forks under mild stress from the trapping of Topoisomerase I cleavage complexes, a damage induction which closely mimics endogenous replicative stress. We observe RAD51 and RAD52, alongside RECQ1, as the first responder proteins to stalled but unbroken forks, whereas Ku and MRE11 are initially recruited to seDSBs. By implementing novel super-resolution imaging assays, we are thus able to discern closely related replication fork stress motifs and their repair pathways.

## Author summary

Damage to the genetic code embedded in an organism’s DNA can result in mutation or cell death which, in turn, can lead to disease and dysfunction. DNA damage is the main cause of many human diseases including cancer, and some forms of neurodegeneration and immune dysfunction. DNA double strand breaks (DSBs), which occur a handful of times in each replicating cell each day, are especially deleterious due to the difficulty of their repair. The main endogenous cause of DSBs is the breakdown of DNA replication forks however there is increasing evidence that damage and stress at these forks can also result in unbroken intermediate structures which avoid DSB formation such as fork regression. We have developed and applied new assays for labelling and visualizing damaged replication forks using super-resolution microscopy. This has enabled us to differentiate between broken and unbroken forks and to discern the different proteins that are recruited for DSB and regressed fork repair. Our data further demonstrate that these assays are widely applicable to DNA damage research and offer a new approach to mapping the spatiotemporal repair of individual damage events inside cells.

## Introduction

**Fig 1:**
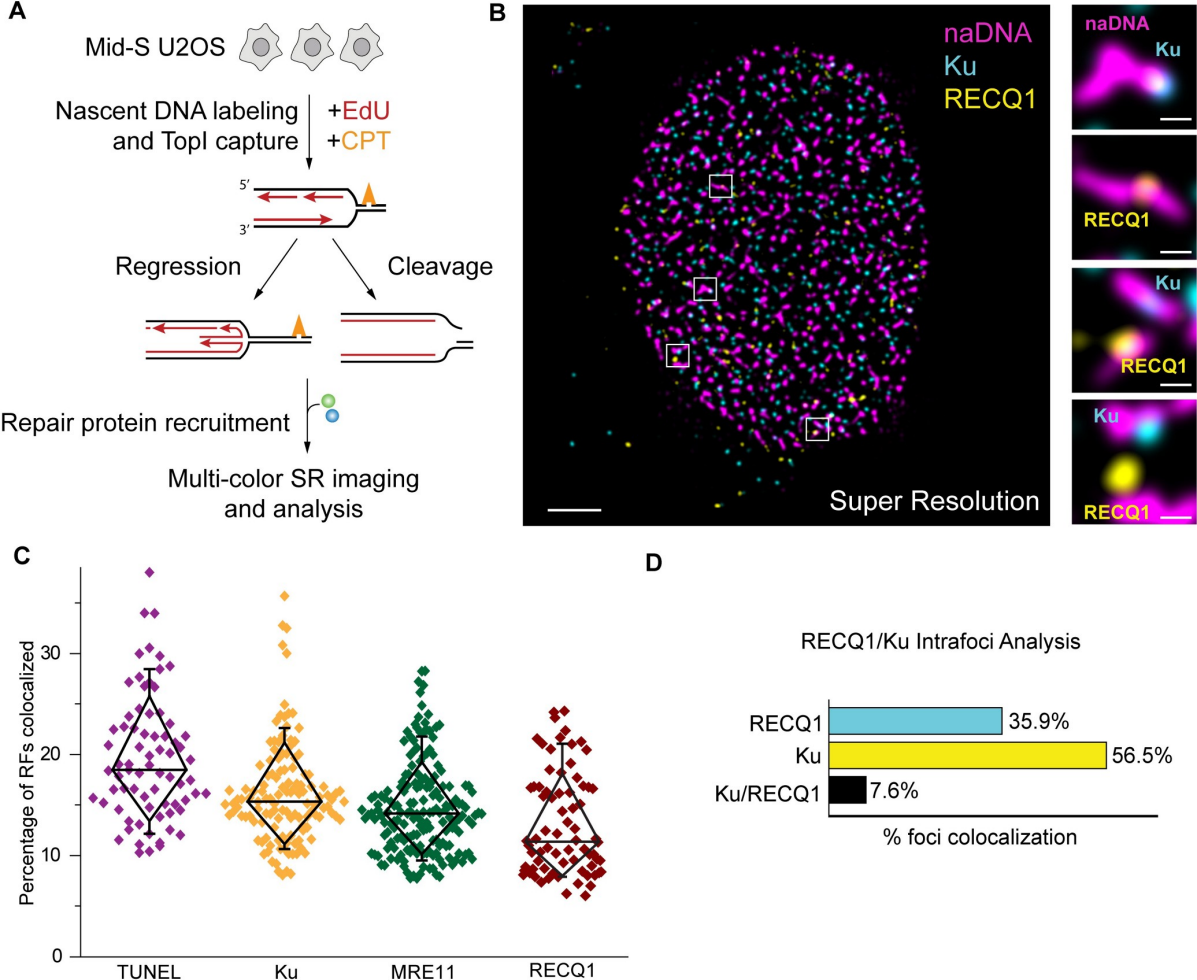
Super-resolution visualization of CPT-induced damaged replication foci. (A) Experimental scheme used to generate and visualize the two hypothesised damage motifs caused by replication stress. (B) Representative super-resolution image of a single nucleus damaged with 100 nM CPT and labelled for naDNA (using EdU, magenta), Ku (cyan) and RECQ1 (yellow). Representative zoomed in images showing either Ku or RECQ1 localized to naDNA foci. Scale bars = 5 μm in whole cell image, 500 nm in zoomed sections. (C) Quantification of the total percentage of RF foci associated with DSBs (via TUNEL signal), Ku, MRE11 and RECQ1 in CPT-damaged cells. (D) Three color analysis showing the exclusionary relationship between RECQ1 and Ku at repair foci. Complete N values available in S1 Table. All graphs show mean ± s.e.m. Student’s t-test results shown for comparison with control levels: ns depicts p>0.05, *** depicts p<0.001, **** depicts p<0.0001.

DNA DSBs are highly genotoxic lesions caused by both exogenous agents and endogenous events related to replication and transcription. Repair of these lesions is predominantly carried out via either non-homologous end joining (NHEJ) or homologous recombination (HR), with the latter pathway requiring a homologous sequence as a template for repair [1,2]. Recently, the existence and conversion of arrested RFs into either regressed ‘chicken foot’ motifs containing a Holliday junction, or single-ended DSBs (seDSBs) (Fig 1A) has been unambiguously demonstrated in higher eukaryotic cells [3–5]. Specifically, it has been shown that upon mild replication stress (for example, clinically induced by Topoisomerase I (TopI) inhibiting anti-cancer drugs), replication forks (RFs) rapidly slow and reverse rather than undergo an inevitable collision with lesions ahead of the fork [6]. This contrasts with another common stressed RF model which involves stalling of the polymerases with continued unwinding resulting in ssDNA generation [7]. This motif can be better mimicked using hydroxyurea [8,9], which depletes deoxyribonucleotides. Conversion of polymerase-stalled RF motifs into regressed forks has not yet been observed and it is understood that conversion of HU-stalled RFs into DSBs requires extended and/or elevated levels of stress [7,9]. This is in contrast to camptothecin (CPT)-stalled RFs, which better mimic endogenous DSB-causing lesions [5]. Which is to say that an individual RF colliding with a single stranded break or physical blockage, can result in a DSB, whereas, depletion of substrates cannot, in and of itself, generate these breaks. For this reason, historically, CPT has been used to generate DSBs whereas mild HU treatment has been used to generate fork stalling, although more recent work has demonstrated the interconnected complexity of the various RF stress motifs [4,10].

With the recent discovery of RF regression in metazoan cells, it is now hypothesized that fork reversal occurs as a specific means, in the event of upstream lesions, to avoid DSB induction, a type of damage that is fundamentally more complicated to repair than damaged but unbroken RFs. In cases of endogenous DSB induction, even in the absence of stress-inducing drugs, lesions ahead of the RF, such as those caused by processes involving single strand break induction including transcription and topographical relaxation, result in characteristic seDSBs that require specific repair by HR [11–14].

A range of proteins has been identified that regulates and contributes to the HR pathway, and a progression of functional interactions has been proposed to describe it [15–17]. Following generation of a DSB, either the HR or NHEJ repair pathway must be selected, and there are clear preferences for one or the other depending on cell cycle status, pathway deficiency, and DSB end chemistry [18–20]. However, the mechanisms of DSB repair pathway choice remain poorly understood [21]. This is in part because interactions between, and competition among, the NHEJ protein Ku and initial HR proteins such as MRE11 (as part of the MRN complex which comprises MRE11-RAD50-NBS1), BRCA1, CtIP, and others have not been definitively characterized *in vivo*. MRN is known to be critical for HR and has been shown to localize immediately to DSB sites with a high affinity for both double stranded DNA and DSBs [22]. This complex is responsible for recruitment of other nucleases, helicases, and mediators, which together orchestrate the extensive resection required at DSBs to generate long 3’ ssDNA overhangs. This resection is an accepted hallmark of canonical HR, and commits the break to HR repair [22,23]. In contrast, the Ku70/Ku80 ring-motif heterodimer (Ku) is understood to cap the DSB end, blocking exonucleolytic resection and stimulating NHEJ repair [24]. How these two responses to DSBs compete and dictate the repair pathway remains unclear, particularly as both MRN and Ku interactions with DSBs are independent of much of the DNA damage response-signaling cascade and lack clear recruitment pathways [2]. MRE11 has also been identified as both an antagonist and a mediator of stalled RF protection, with some studies demonstrating BRCA2- and PARP1-dependent RF protection from MRE11 degradation [8,25,26]. Conversely, others have argued that recruitment of PARP1 by MRE11 is required for reparative resection [27].

A limiting factor in defining the internal organization and repair progression at stalled/regressed and broken RFs *in vivo* has been the spatial resolution of fluorescence microscopy, a limitation imparted by the diffraction of electromagnetic radiation [28]. This has made it exceptionally difficult to distinguish between single and clustered repair sites, as well as the many proteins involved. Indeed, conventional immunofluorescence imaging routinely requires upwards of 100 colocalized fluorophores to generate discernable foci within the nucleus [29], often necessitating the use of clustered or overly potent damage induction. Single molecule localization SR imaging confers a ten-fold improvement in image resolution over conventional fluorescence microscopy [28,30] together with single-molecule sensitivity, and thus affords the possibility of examining single DSB sites *in vivo* [31]. Recently, we developed and applied novel single molecule super resolution (SR) imaging to visualize several of the key steps of the homologous recombination repair of seDSBs [31]. This study involved the spatiotemporal mapping of resection and RAD51/ssDNA nucleofilament formation over 16 hours recovery from CPT-induced seDSB formation. Building on this research, here we set out to design and implement new SR assays to probe the earlier stages of RF stress, and, specifically, to differentiate stalled/regressed RFs from broken DSBs. This constituted a natural continuation of our previous work because several of the proteins involved in HR have recently been hypothesized to play protective, reparative, or even antagonistic roles at unbroken but stalled/regressed RFs based on biochemical and electron microscopy experiments [26,32–34].

Utilizing novel SR imaging assays, we are able to specifically visualize the initial recognition processes of stressed and broken RFs by first responder proteins, and differentiate between these distinct damage motifs. We observe the regressed RF repair helicase RECQ1 is recruited to stalled but unbroken forks within the first 60 minutes of damage response alongside the critical HR recombinase RAD51, and its less well-understood binding partner RAD52. This timing contrasts with the eventual recruitment of RAD51, RAD52 and BRCA2 (another critical HR protein) to resected DSBs to form the long single stranded DNA/RAD51 nucleoprotein filament which undertakes homology search and strand invasion. We previously shown that this RAD51/RAD52 interaction occurs only following 2–4 hours of resection [31]. We also find that DSBs recruit both Ku and MRE11 irrespective of their eventual repair pathway choice and do so in a spatially distal organization such that Ku interacts with the DSB end and MRE11 with nearby double-stranded DNA. PARP-inhibition successfully blocks stalled fork protection, converting these damage structures into seDSBs. Our data represent the first observation and differentiation of individual stalled RFs from seDSBs *in vivo* and highlight the potential of single molecule SR assays for interrogating sub-populations of damage motifs within in cells. In applying these assays we are able to describe the behaviors of first responder proteins at stalled RFs and seDSBs at low levels of genomic stress.

## Results

### SR assays enable visualization of distinct replication-associated DNA damage events

We used multicolor single molecule SR microscopy [28,30,35–37] to determine the spatiotemporal behavior and crosstalk of key ‘first responder’ proteins at individual damaged RFs in human cells (Fig 1A and 1B). To achieve this, we synchronized U2OS cells in early-mid S-phase and induced a low level of replication stress by treating them with 100 nM camptothecin (CPT) for one hour [5,12]. Concurrently, we pulse-labelled nascent DNA (naDNA) using EdU to detect all active RFs [17,37,38]. We considered the potential for this simultaneous damage/labelling approach to result in a loss of EdU incorporation in response to damage to all RFs, or cell cycle arrest. Comparison of CPT-treated and control cells, however, did not demonstrate any loss of EdU signal (S1 Fig) and we further concluded that only a minor proportion of RFs were affected by the low level of CPT damage (Fig 1C). This was in good agreement with previous reports of low DSB induction at similar doses [5,6]. Furthermore, our observations support recent work that shows that individual RFs exist within the cell spatially distinct from each other, with only pairs of RFs from divergent origin firings forming significant higher order structures during early-mid S phase [39,40]. In our hands, hundreds of spatially distinct EdU foci are visualized in each z-slice of a nucleus (Fig 1B, S2A–S2E Fig). When visualizing damage or replication markers, their immunolabelled signal usually presents as a single punctum overlapping the larger EdU focus. This indicates that there are not distinct damage or replication locations associated with the replication foci (i.e. not multiple forks/seDSBs). We cannot discount the possibility that multiple damage or replication events co-exist below the spatial resolution of our images (<40–50 nm), however we consider this unlikely and also highlight the characteristic arrangement of the replication factors MCM and PCNA within these individual replication foci. Not only are these factors found on the periphery of the EdU foci, but they are correctly ordered with MCM ahead of the PCNA (SI1A-C). Further demonstrating the strength of our SR imaging assays, we have established that the information obtained in 2D SR projections is representative of, and did not alter conclusions from, flattened 3D SR images (S2D–S2K Fig).

At low dosages, CPT captures TopI cleavage complexes ahead of a subpopulation of progressing RFs; this causes them to slow, stall and potentially regress or break to form seDSBs [5]. As we have reported previously, by labeling naDNA using pulsed EdU incorporation we are able to visualize CPT-damaged RFs [17,31]. Apart from advantageously positioning DSBs at naDNA foci, CPT is an ideal drug for studying the HR process because the resulting seDSB products closely resemble those generated by endogenous stress from RF-competitive transcription and topographical modification activity [5,10]. To provide spatiotemporally resolved colocalization information, we also developed and validated co-staining assays optimized for SR imaging based on immunolabeling protocols for visualizing key DNA repair proteins, and a modified Terminal deoxynucleotidyl transferase dUTP nick end labeling (TUNEL) assay for enzymatically labelling DSBs [41]. The TUNEL assay has previously been widely used to detect apoptotic DNA fragmentation by post-fixation addition of fluorescently visualizable markers to 3’-hydroxyl termini using deoxynucleotide transferase. Because of the single molecule sensitivity of our SR imaging, we were able to visualize individual DNA DSBs directly after addition of deoxynucleotides conjugated to a distinct fluorophore.

By combining these labelling approaches, we developed a set of assays to robustly and reproducibly generate three-color SR images of z-slices of single nuclei in which hundreds of single replication foci were distinguished and various colocalized proteins quantified. To test the strength of our approach, we labeled DSB ends using the TUNEL assay and immunolabelled several key proteins that have been identified as potential first responders in different fork repair pathways, including Ku, MRE11, RAD51, RAD52 and TOPI [42–45]. To specifically detect stalled RFs, we immunolabelled RECQ1 helicase, because it has recently been shown to be involved in rescue of stalled but unbroken RF motifs but has no defined role in DSB repair [43,44]. Immunostaining of Ku and RECQ1 within the same cells immediately revealed an exclusionary relationship in which these two proteins were observed to minimally colocalize with each other at naDNA foci (Fig 1B). Recent literature has demonstrated Ku’s affinity for blunt DSB ends *in vivo* and *in vitro* [24] and it is increasingly clear that Ku can load at DSBs irrespective of the eventual repair pathway [45,46]. In conjunction with our finding that Ku did not colocalize with RECQ1, a known regressed RF repair helicase [44], we identified Ku as a potential marker for seDSBs but not stalled RFs. As a preliminary calculation we quantified the percentage of replication foci colocalized with each of Ku, RECQ1, and MRE11, a protein with hypothesized roles at both seDSB [47] and stalled RFs [25,26]. We also quantified the percentage of replication foci colocalized with the TUNEL signal, our presumptive direct marker for a DSB (Fig 1C). These calculations confirmed that only a minority of RFs had been affected by the low dosage of CPT, with between 10 and 30% of replication foci positive for a damage marker (Fig 1C). Furthermore, by only considering naDNA foci that colocalized with either Ku, RECQ1, or both we were able to exclude unaffected RFs (~75%) and determine that indeed very few damaged naDNA foci colocalized with both Ku and RECQ1 (7.6%) (Fig 1D). This clearly demonstrated the specificity of these two proteins (and their respective antibodies) for distinct damage motifs (i.e. Ku is only recruited to seDSBs while RECQ1 is only recruited to regressed, unbroken RFs) and showed that our novel SR imaging assays could successfully discern individual regressed RFs from seDSBs *in vivo*, something that has not been achieved previously.

Unexpectedly, the percentage of RFs colocalized with MRE11 was lower than those colocalized with Ku, which appeared to contradict the hypothesis that MRE11 would be detected at both regressed and broken RFs. TUNEL labelling yielded the highest level of occurrence, potentially indicating that some DSBs had recruited neither Ku nor MRE11. We considered this an unlikely possibility because without Ku/MRE11 recruitment a DSB would be left unrepaired resulting in gross genomic instability. We reasoned that this observation was more likely due to limitations of the antibody staining, in particular under-staining of individual proteins due to steric hindrance of the antigen, and possible under-sampling because of the super-resolution modality. In contrast, the TUNEL assay potentiated a gold-standard for direct DSB detection because of the assays long incubation time (12 hours) and the comparatively smaller size (< 3 nm) of the fluorophore conjugate, minimizing steric exclusion. We set out to further test and standardize this novel assay.

To achieve this, we aimed to better quantify the different naDNA/protein/TUNEL association events such that we mitigated the potential for over-estimation of colocalization due to the densely populated nuclear environment and cell-to-cell differences in total signal densities. We used our recently established analytical protocol for quantification of nuclear colocalization by normalizing the number of detected overlaps to the number of overlaps due to random colocalization [48]. For each single 2-color nucleus image, the naDNA and TUNEL/protein foci were defined using automated particle analysis and then the foci in one channel were randomly redistributed throughout the nuclear region of interest (ROI) using a Monte Carlo simulation. The number of overlaps detected in the real nucleus image could then be ratioed to the average number of overlaps detected in the random simulations (taken from 20 simulations) to give a value of 1 if the real-image associations were random and a value above 1 if there was more-than-random colocalization. Based on this analysis, a value of 2 indicated twice as many colocalizations as compared to random, a value of 3 indicated triple, and so on. Importantly, this successfully normalizes cell-to-cell differences in nuclear size as well as the number, size and density of clusters. Furthermore, even if hundreds of colocalizations are present because of the high density of the image, the random simulations will return this same number and the colocalization will be determined insignificant. Such analysis also allows comparison of colocalization in CPT-treated cells with both control levels detected in healthy replicating cells and random levels in simulated data (For details of this and associated analytical protocols for SR data see S3 Fig and [31,48]).

**Fig 2:**
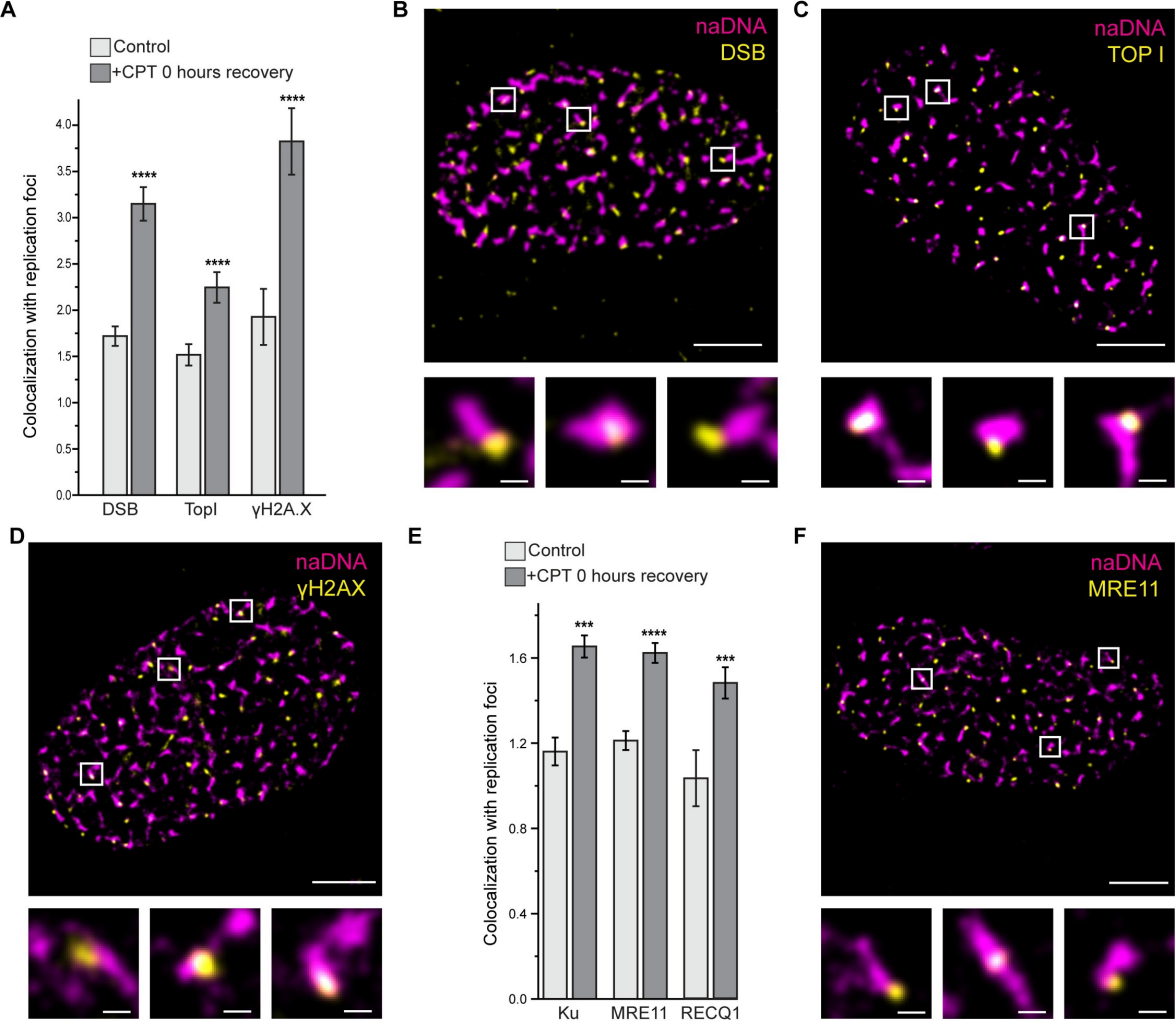
Both fork protection and HR repair proteins colocalize with replication foci following CPT-induced damage. (A) Quantification of colocalization of EdU labelled replication foci (naDNA) with DSBs (via TUNEL labelling), TopI, and γH2AX in undamaged and CPT-damaged cells. (B) Representative SR image of a single nucleus damaged with 100 nM CPT and labelled for naDNA (magenta) and DSBs (using TUNEL)(yellow). Zoomed in regions show colocalization. Representative undamaged whole-cell image shown in S7A Fig. (C) Representative SR image of a single nucleus damaged with 100 nM CPT and labelled for naDNA (magenta) and TopI (yellow). Zoomed in regions show colocalization. Representative undamaged whole-cell image shown in S7B Fig. (D) Representative SR image of a single nucleus damaged with 100 nM CPT and labelled for naDNA (magenta) and γH2AX (yellow). Zoomed in regions show colocalization. Representative undamaged whole-cell image shown in S7C Fig. (E) Quantification of colocalization of replication foci (naDNA) with Ku, MRE11, and RECQ1 in undamaged and CPT-damaged cells. Representative whole-cell control and CPT-treated cells for naDNA/Ku and naDNA/RECQ1 labelling shown in S7E–S7H Fig. (F) Representative SR image of a single nucleus damaged with 100 nM CPT and labelled for naDNA (magenta) and MRE11 (yellow). Zoomed in regions show colocalization. Representative undamaged whole-cell image shown in S7D Fig. Complete N values available in S1 Table, number of replicates >3, number of cells imaged typically 20–60. All graphs show mean ± s.e.m. Student’s t-test results shown for comparison with control levels: *** depicts p<0.001, **** depicts p<0.0001. Scale bars = 3 μm in whole cell images, 250 nm in zoomed sections.

Using this analysis to further test our labelling assays, we quantified replication foci colocalization with TUNEL, the phosphorylated histone damage marker γH2AX, and TopI signal and found that in all cases colocalization increased significantly following CPT damage (Fig 2A–2D). The above-random overlap of γH2AX in control cells was expected, particularly as we used U2-OS cells which are prone to increased endogenous replication stress and heightened γH2AX levels [49]. TopI association with undamaged RFs occurs due to its role relaxing DNA supercoiling ahead of progressing forks [6]. Significantly higher than random colocalization of TUNEL signal with replication foci was also detected in control cells leading us to hypothesize some degree of non-specific TUNEL labelling at unbroken RFs. We note, however, that TUNEL colocalization did increase to more than triple the amount of random colocalization upon CPT treatment, confirming that the majority of TUNEL-labelled foci were specific for seDSBs (Fig 2A and 2B).

In contrast to the TUNEL and γH2AX images, the associations of Ku, MRE11 and RECQ1 with naDNA in undamaged control cells were only slightly more than calculated in random simulations, and indeed, equivalent to random localization in the case of RECQ1 (Fig 2E and 2F). The small amount of MRE11 and Ku colocalization could be explained by the transient binding of these proteins, both of which have affinities for ssDNA, nicks, and gaps that are not associated with DSBs [50,51]. This result revealed only minimal association between repair proteins and naDNA in undamaged cells and demonstrates the sensitivity of our colocalization analysis. Importantly, quantification of the association of Ku, MRE11 and RECQ1 in CPT-treated cells demonstrated the same significant increases in colocalization as for DSBs, TOPI and γH2AX, and as was expected in response to fork stalling and seDSB induction (Fig 2).

### Ku and MRE11 are contemporaneous and persistent first responders to seDSBs

Ku’s comparable level of recruitment with MRE11 to seDSBs was somewhat surprising given their opposing and competitive roles in DSB repair and our historical view that Ku loading at DSBs initiates repair via NHEJ and blocks recruitment of HR proteins [52,53]. However, several recent studies have found evidence that on short timescales Ku can initially be loaded at DSBs later repaired by HR [46] and, specifically, to seDSBs. However given the one hour of CPT-damage and evidence that HR nucleases actively remove Ku [45], we expected to detect lower levels of Ku colocalization. Importantly, it is well documented that CPT-treatment generates seDSBs that are repaired by HR because, as well as generating these breaks specifically in S-phase, the single-ended nature of the breaks precludes ligation to an opposing DSB end. Thus, we concluded that we were observing persistent Ku recruitment to DSBs that would, nonetheless, ultimately be repaired by HR.

In order to focus on the first responder proteins, we confirmed the temporal progression of Ku, MRE11, and RAD51 so as to separate the key canonical steps involved in HR–break recognition, resection, RAD51 nucleoprotein filament formation, homology search and repair. In doing this we aimed to demonstrate that our observations immediately following one hour of CPT-treatment were specific for early HR. We found that although Ku was initially present at seDSBs, within 60 minutes of recovery (and thus potentially 2 hours after break induction) it had been removed (S3A Fig). In contrast, MRE11 was recruited to repair foci and remained strongly associated for the first 2 hours of repair during which time we have previously shown that naDNA is resected to generate ssDNA (as detected using BrdU incorporation and RPA colocalization [31]) (S4B and S4C Fig). Coinciding with resection, association of RAD51 was detected most strongly at 2–4 hours at which time the RAD51/ssDNA nucleofilament was successfully formed prior to homology search (S3C Fig). 12 hours after damage induction, we observed that RAD51 levels had returned almost to control levels, providing an approximate timescale for *in vivo* HR repair [31].

These data confirmed that our observations of Ku and MRE11 localization to replication foci immediately following CPT treatment were indicative of initial damage recognition processes. Next we sought to determine the specificity of the TUNEL assay for seDSBs because, although we detected an large increase in TUNEL signal following CPT-treatment, we hypothesized that some of this signal could be due to stressed RF species, in particular from stalled and regressed replication forks, because of the high level of colocalization detected in undamaged cells (Fig 2A) [5,6]. This would present a confounding variable in our use of the TUNEL assay as a seDSB-specific marker for probing the spatial and interdependent relationships of proteins at RF-associated DSBs. Demonstrating that this assay could differentiate between stalled and broken forks would allow future investigation into the key underlying mechanisms of CPT damage–and, indeed, of endogenous DSB-inducing replication stress, namely that the cell employs various complex mechanisms by which to avoid RF collision resulting in DSBs. Previously, it has been shown that this involves slowing, or complete stalling, of the RF (which has traditionally been visualized using pulse/chase DNA fiber analysis) and reversal of the fork to generate a four-way junction (has more recently been shown using electron microscopy) [3,6,42,43]. These stressed RF motifs do not harbor a DSB and so are, presumably, more easily repaired, however, the regressed arm of a RF presents as a blunt end which could, conceivably, be detected by the TUNEL assay and prove difficult to distinguish by SR.

**Fig 3:**
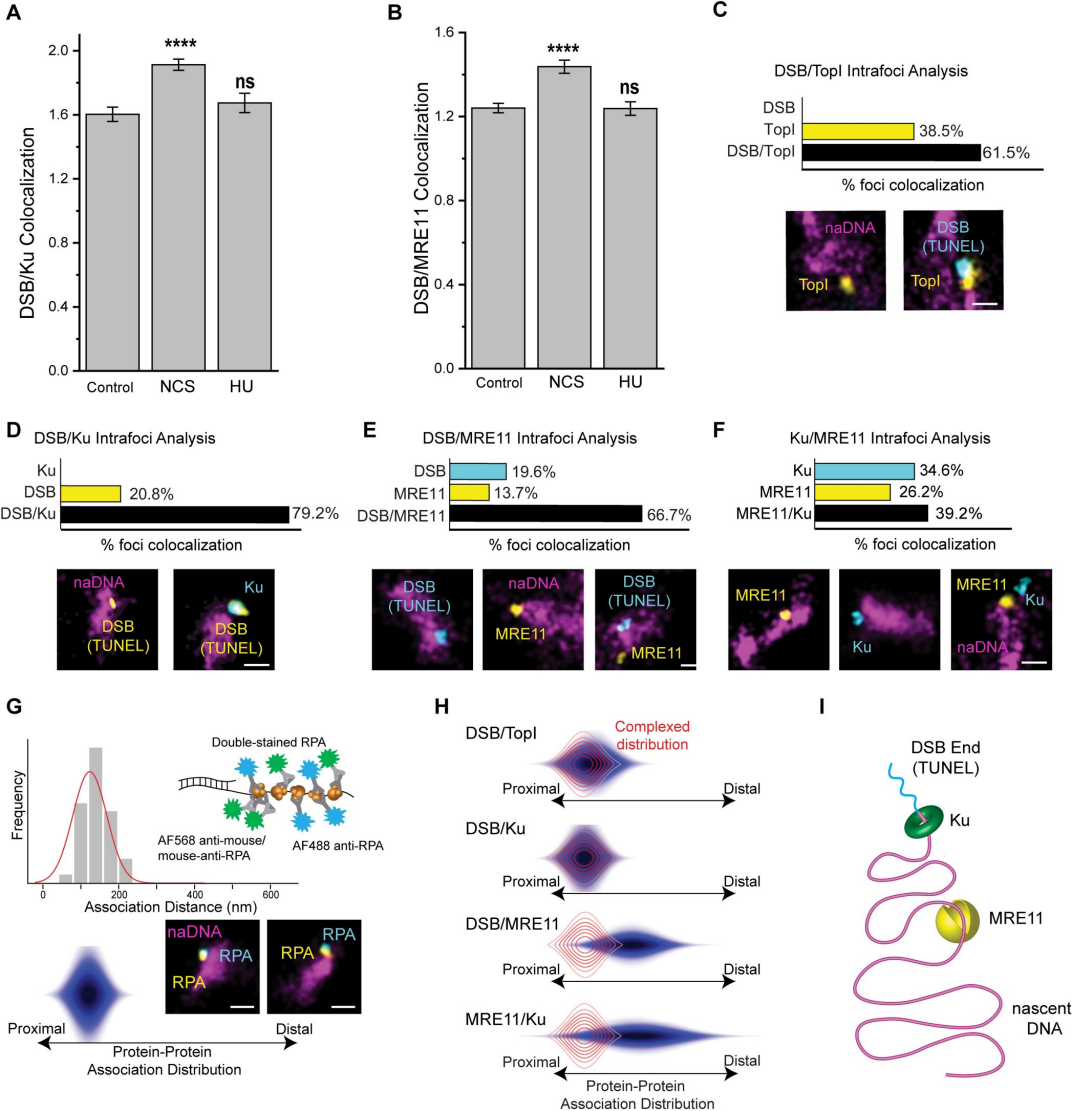
Ku and MRE11 colocalize to seDSB repair foci in a distally separated arrangement with Ku associating with the DSB end and MRE11 with nearby naDNA. (A) Quantification of colocalization of DSBs (via TUNEL labelling) with Ku in control cells and in cells immediately following treatment with CPT, NCS or HU. Representative whole-cell images shown in S8A–S8C Fig. (B) Quantification of colocalization of DSBs (via TUNEL labelling) with MRE11 in control cells and in cells immediately following treatment with CPT, NCS or HU. Representative whole-cell images shown in S8D–S8F Fig. (C) Three color analysis of TopI and DSB (using TUNEL labeling) localization at repair foci immediately following 1 hour of CPT damage. Representative whole-cell control and CPT-treated cells shown in S9A and S9B Fig. (D) Three color analysis of Ku and DSB (TUNEL) localization at repair foci immediately following 1 hour of CPT damage. Representative whole-cell control and CPT-treated cells shown in S9C and S9D Fig. (E) Three color analysis of MRE11 and DSB (TUNEL) localization at repair foci immediately following 1 hour of CPT damage. Representative whole-cell control and CPT-treated cells shown in S9E and S9F Fig. (F) Three color analysis of MRE11 and Ku localization at repair foci immediately following 1 hour of CPT damage. Representative whole-cell control and CPT-treated cells shown in S9G and S9H Fig. (G) A histogram (top) and resulting 3D color-contoured distribution (bottom) and accompanying model (right) showing quantification of the rendered distance between the centers of mass of the same super-resolved RPA foci immunolabeled indirectly with mouse-anti-RPA/Alexa-568-anti-mouse and directly with Alexa-488 conjugated rabbit anti-RPA. These distributions depict proteins closely associated within the foci because of RPA accumulation on ssDNA. (H) Color-contoured protein-protein association distribution maps of protein associations immediately following CPT damage. The red overlap distribution shows the RPA/RPA modelled proximal distribution. (I) A model depicting the association of Ku and MRE11 with a seDSB repair foci in which the TUNEL signal localizes to the break alongside KU while MRE11 localizes distally to double-stranded naDNA. Representative foci shown, scale bars represent 250 nm. Complete N values available in S1 and S2 Tables, number of replicates >3, number of cells imaged typically 20–60, number of individual foci manually scored typically 50–70.

Thus, to establish the specificity of the TUNEL assay and to further verify the seDSB-specific detection of our assays, we induced two distinct types of DNA damage, by treating cells with either neocarzinostatin (NCS, 200 ng/ml, 1 hour), which generates two-ended DSBs without inducing replication stress [54], or with a low dose of hydroxyurea (HU, 1 mM, 4 hours), a small molecule drug that causes replication stalling but minimal DSBs under the conditions used [9,34]. In the case of NCS damage, we were unable to use naDNA as a marker for potential damage sites because NCS-induced DSBs are randomly distributed throughout the nucleus, and are not RF specific. Instead we quantified the colocalization of TUNEL signal with MRE11 and Ku immediately following damage and detected a significant increase demonstrative of NCS-induced DSBs (Fig 3A and 3B). In contrast, quantification of TUNEL association with both Ku and MRE11 in HU treated cells showed no significant increase which would have indicated off-target TUNEL labelling of stalled RFs and their association with Ku/MRE11 (Fig 3A and 3B). Furthermore, no colocalization between TUNEL and RECQ1 was detected in NCS-damaged cells (S5A Fig). Interestingly a persistent non-random level of TUNEL and TUNEL-associated MRE11 and Ku existed within undamaged cells, possibly due to genuine endogenous levels of genomic stress which are known to be elevated in cancer cells [55,56].

### Colocalized Ku and MRE11 at seDSBs are distally separated on the DNA

Confident that the TUNEL assay specifically labelled DSBs and not stalled or regressed RFs, and that by fixing cells immediately after one hour of CPT treatment we were visualizing the initial break recognition steps of HR repair, we endeavored to elucidate further spatiotemporal detail. Analysis of three-color images in which both DSBs (via TUNEL) and TopI were labeled alongside naDNA showed that DSB localization to the RF is dependent on the presence of TopI (Fig 3C). This result was determined by quantifying the percentage of naDNA foci that were positive for only one or both of the two signals (S3 Fig).

Demonstrating the dependence of DSB formation on TopI, we found no colocalization between DSBs with replication foci in the absence of a coinciding TopI signal. In contrast, 38.5% of naDNA-associated TopI was detected without a colocalized DSB, likely because of TopI either trapped or transiently bound ahead of unbroken RFs (Fig 3C). 61.5% of TopI-positive replication foci were also positive for a TUNEL signal, showing a strongly dependent colocalization. Together, these observations demonstrate successful induction of DSBs due to CPT captured TopI complexes colliding with active replication forks.

Next, we considered Ku and MRE11 and their interplay at DSBs. While MRE11 is an integral resection mediator necessary for HR of DSBs, Ku is a core NHEJ protein and its fast unloading and exclusion from HR-fated seDSBs has previously been shown [57,58]. Nevertheless, we note that the unloading of Ku after one hour is in good agreement with its natural dissociation rate [59] and with recent observations [60] including the blocking of Ku reloading onto minimally resected DNA ends [61].

To investigate the crosstalk of MRE11 and Ku at DSBs, we assessed three-color images to discern their interplay at repair foci. Both MRE11 and Ku were found to colocalize strongly with naDNA associated seDSBs marked by TUNEL, with 66.7% of all Ku positive RFs displaying a DSB signal alongside Ku, while 79.2% of the naDNA foci colocalized with either a DSB or MRE11 demonstrated the coexistence of MRE11 alongside the DSB. Only 20.8% of all DSBs detected lacked a colocalized Ku signal, whereas 19.6% lacked an MRE11 signal. Ku was not detected at naDNA foci in the absence of a break, and only 13.7% of MRE11 association was with RFs lacking a DSB signal (Fig 3D and 3E). When MRE11 and Ku were co-stained, they were found to associate with repair foci both individually (26.2% and 34.6%, respectively) and together (39.2%), convincingly demonstrating that neither MRE11 nor Ku localization at a single DSB precluded association with the other (Fig 3F).

Because of the high degree of colocalization of Ku with MRE11 at repair foci and their contrary roles in repair pathway choice, we set out to define the internal spatial arrangement of these key proteins within single repair foci by taking advantage of the ten-fold enhanced resolution of our images. To do this, we modelled two-color SR images of closely associated proteins by dual-labelling RPA with Alexa Fluor 568 and 488 in fixed, CPT-damaged cells (Fig 3G) [31]. We then determined the lateral offsets between the two different color signals for more than 50 foci to generate a histogram and 2D protein-protein association distribution map. Because the two different immunolabels of RPA target the same underlying RPA focus, the detected lateral offsets (defined as the protein-protein association distribution) represent protein-protein colocalization that is predominantly proximate and potentially interacting. By similarly determining the distribution maps of pairs of proteins, or DSBs and proteins, colocalized with repair foci, we were able to establish whether they were proximally or distally associated with one another (S3C Fig).

The DSB-TopI association distribution was found to be narrow and spatially proximal, matching closely the distribution determined for dual-stained RPA (Fig 3H, red complexed distribution shown from 3G). This confirmed that DSBs occur close to CPT-trapped TopI, and that collision between RFs and single stranded lesions are the predominant cause of the induced DSBs. Similarly, the DSB-Ku spatial distribution demonstrated a close proximal association due to the loading of Ku onto the broken DNA end. In contrast, analysis of the association distribution between both DSBs and MRE11, and MRE11 and Ku, demonstrated distal arrangements in which MRE11 associated with naDNA within the repair foci but away from the seDSB site and Ku (Fig 3H and 3I). Thus, although MRE11 and Ku routinely colocalize at the same DSB focus, Ku associates directly with the DSB end, while MRE11 localizes distally to naDNA away from the break. This observation is consistent with the endonuclease activity of MRE11, but contradicts current models in which MRE11, as part of the MRN complex, interacts with dsDNA close to the DSB site [18,46,47].

### Stalled RFs recruit RAD51 and RAD52 for protection and repair

**Fig 4:**
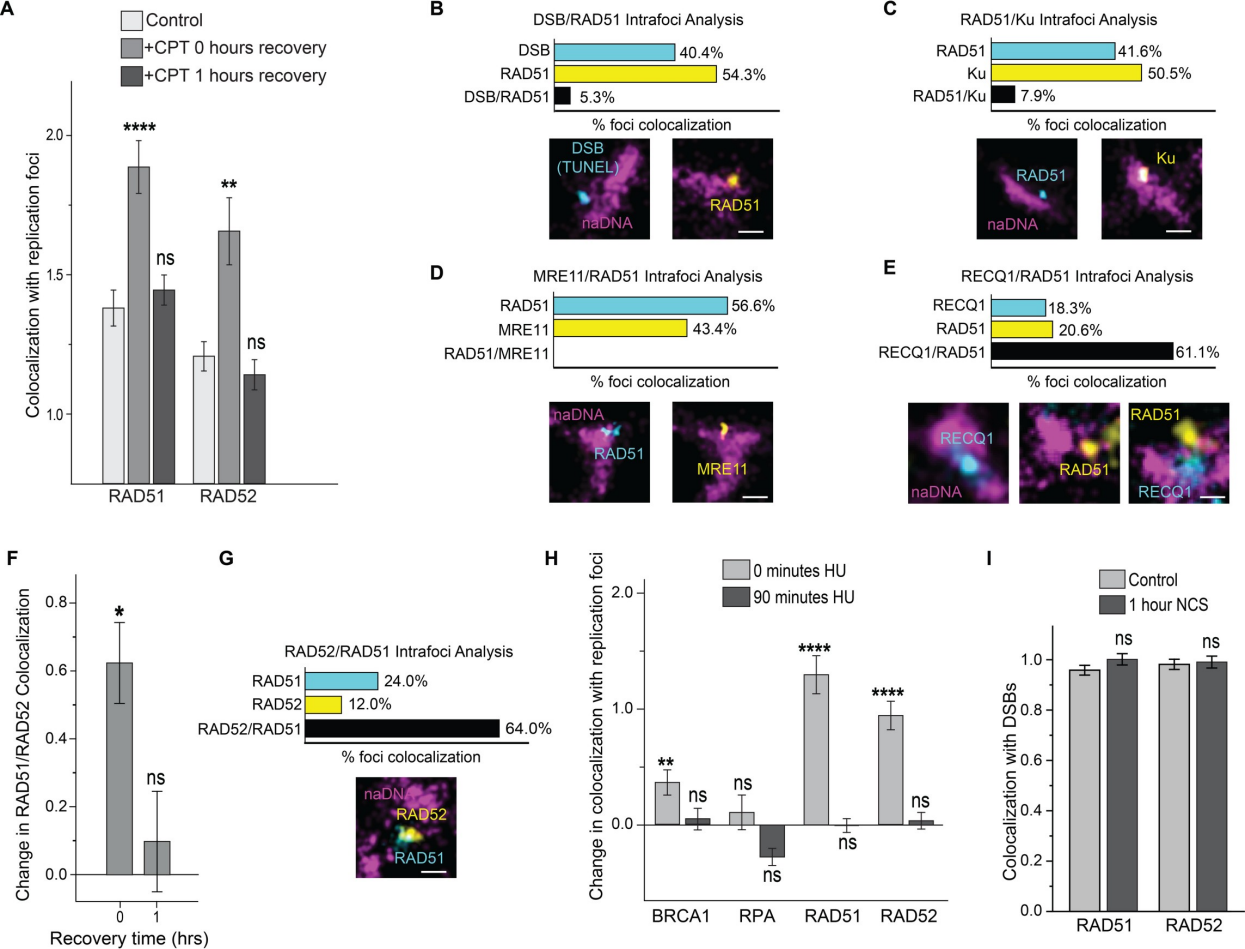
RAD51 and RAD52 are first responders to stalled/regressed RFs. (A) Quantification of RAD51 and RAD52 colocalization with RF foci immediately after CPT damage and following 1 hour of recovery. Representative whole-cell control and CPT-treated cells shown in S10A and S10B Fig. (B) Three color analysis of RAD51 and DSB (TUNEL) localization at RF foci immediately following 1 hour of CPT damage. Representative whole-cell control and CPT-treated cells shown in S10C and S10D Fig. (C) Three color analysis of RAD51 and Ku localization at RF foci immediately following 1 hour of CPT damage. Representative whole-cell control and CPT-treated cells shown in S10E and S10F Fig. (D) Three color analysis of RAD51 and MRE11 localization at RF foci immediately following 1 hour of CPT damage. Representative whole-cell control and CPT-treated cells shown in S10G and S10H Fig. (E) Three color analysis of RAD51 and RECQ1 localization at RF foci immediately following 1 hour of CPT damage. Representative whole-cell control and CPT-treated cells shown in S10I and S10J Fig. (F) Quantification of colocalization of RAD51/RAD52 immediately following 1 hour of CPT treatment and after 1 hour of recovery in normal medium, normalized to control levels. (G) Three color analysis of RAD52 and RAD51 localization at RF foci immediately following 1 hour of CPT damage. Representative whole-cell control and CPT-treated cells shown in S10A and S10B Fig. (H) Quantification of protein colocalization with naDNA RF foci at unbroken HU-stressed RFs immediately following 4 hours of HU treatment and after 90 minutes of recovery in normal medium from HU treatment, normalized to control levels. (I) Quantification of RAD51 and RAD52 colocalization with DSBs (TUNEL signal) in control and NCS-damaged cells. Representative foci shown, scale bars represent 250 nm. Complete N values available in S1 and S2 Tables, number of replicates >3, number of cells imaged typically 20–60, number of individual foci manually scored typically 50–70. All graphs show mean ± s.e.m. Student’s t-test results shown for comparison with control levels: ns depicts p>0.05, * depicts p<0.05, ** depicts p<0.01, **** depicts p<0.0001.

Although the expected temporal progression of break recognition, resection, and RAD51/ssDNA nucleofilament formation was largely reflected in our data (S4 Fig), we were surprised to detect small, yet statistically significant, accumulations of RAD51 at replication foci within 60 minutes of CPT treatment (Fig 4A). Even more surprisingly, the observed low levels of RAD51-naDNA association returned to control levels after a further 60 minutes of recovery. This return to control levels occurred before RAD51 associations detected at 2–8 hours recovery (S4 Fig). We have previously characterized these later time-points, confirming that RAD51 detection after 2 hours is indicative of resection having occurred allowing for construction of the RAD51-ssDNA nueclofilament prior to homology search [31]. While RAD51 is a key protein in HR, nucleofilament formation and recombinase activity have been shown to occur only after extensive resection and RPA coating [23]. Because of this, RAD51 foci formation is usually detected 2–8 hours following DSB induction and nucleofilament formation is not a reasonable explanation for our detection of RAD51 immediately following 1 hour of CPT treatment [62–65]. Moreover, despite a significant increase in colocalization with RF foci immediately following CPT treatment, this could not have represented nucleofilament formation because of its return to control levels after 60 minutes of recovery. In light of recent reports on the interplay of RAD52 with RAD51, we also quantified RAD52 localization with naDNA foci and found that it followed a similar pattern to RAD51 including fast recruitment before dissipating after one hour. (Fig 4A).

To further elucidate the role of the rapidly recruited RAD51 and RAD52, we analyzed three-color images in which RAD51 was co-stained with naDNA and TUNEL-labeled DSBs. This revealed that RAD51 was excluded from DSB positive naDNA, with a greater number of RAD51-positive RF foci (54.3%) than DSB-positive foci (40.4%), but minimal colocalization of RAD51 and DSBs (5.3%) (Fig 4B). Similarly, co-staining of RAD51 with either Ku or MRE11 revealed either minimal (7.9%) or no colocalization at replication foci, respectively (Fig 4C and 4D). We interpret these observations to mean that while Ku and MRE11 colocalize strongly with replication foci harboring seDSBs, the early RAD51 and RAD52 association with replication foci immediately following CPT treatment entails recruitment independent of break formation and canonical HR repair. Strikingly, we found significant colocalization between RAD51 and RECQ1, implicating that the detected early RAD51 recruitment occurs at stalled RFs (Fig 4E).

Despite a lack of well-established role in HR, mammalian RAD52 forms foci during repair and colocalizes with RAD51 foci during late recombinase activity [65,66]. More recently, we demonstrated that *in vivo* RAD52 acts as the key mediator of early ssDNA/RAD51 interaction, but that it can be replaced by BRCA2 without functional consequence [31]. We therefore considered the possibility of a similar RAD51/RAD52 association and function independent of HR. To test this hypothesis we quantified the change in colocalization between RAD51 and RAD52 immediately following damage and after one hour of recovery, and detected an increase in colocalization at the 0 hour time point but not after 1 hour of recovery, at which time it was found to be at the same level as in control, undamaged cells. This was in agreement with the trends of both proteins’ independent associations with naDNA (Fig 4F). Three-color analysis of these foci confirmed that RAD51/RAD52 colocalized together at naDNA foci immediately following CPT damage (Fig 4G). Based on these observations, we determined that RAD51/RAD52 recruitment to replication foci, while still a consequence of CPT-induced replication stress, occurred specifically at unbroken forks that have stalled or regressed [6,9,32,43].

To further demonstrate this unexpected differentiation in first responder proteins between stalled and broken RFs we again treated cells with HU and assessed colocalization of RAD51, RAD52, RPA and BRCA1 with naDNA [67]. Cell were treated with 1 mM of HU for 4 hours, resulting in replication stress and fork stalling, but minimal fork breakage [9]. Under these conditions we found that RAD51, RAD52 and BRCA1 all localized to RFs (Fig 4H), but upon HU removal, this association diminished within 90 minutes, demonstrating a much faster repair process than canonical HR, which takes several hours. Importantly, we did not detect increased RPA at these foci at either 0 or 90 minutes of recovery following HU treatment, showing the lack of extensive resection required for repair of stalled but unbroken RFs. Thus, notwithstanding questions concerning the role of RAD52 [68–71], these observations supported our hypothesis that both RAD51 and RAD52 are recruited to stalled/regressed RFs.

To contrast HU treated cells, we also assessed cells treated with NCS to generate DSBs in the absence of RF stress. In these cells we found no increased association between DSBs and either RAD51 or RAD52 immediately following one hour of NCS damage (Fig 4I) further demonstrating the early RAD51/RAD52 recruitment to DSBs is uniquely associated with RF stalling/regression. Finally, we assessed early MRE11 association with RAD51 across all drug treatments used here and found no evidence of colocalization, once again revealing their mutually exclusive roles at seDSBs and stalled RFs immediately after low levels of damage induction (S4B Fig).

### CPT treatment combined with PARP-inhibition synergistically converts stressed RFs into seDSBs

It has previously been reported that RF protection by stalling/regression is PARP-dependent [6] and more recently, it was discovered that PARP inhibition causes DNA lesions to go unrecognized, blocking the consequent RF stalling/regression that enables repair, instead causing an increase in seDSBs induction [72]. In light of these findings we reasoned that inhibiting PARP in cells that are treated with low dosage of CPT would cause the resulting RAD51/RAD52-positive stalled RFs to be bypassed to generate more TopI-induced seDSBs.

**Fig 5:**
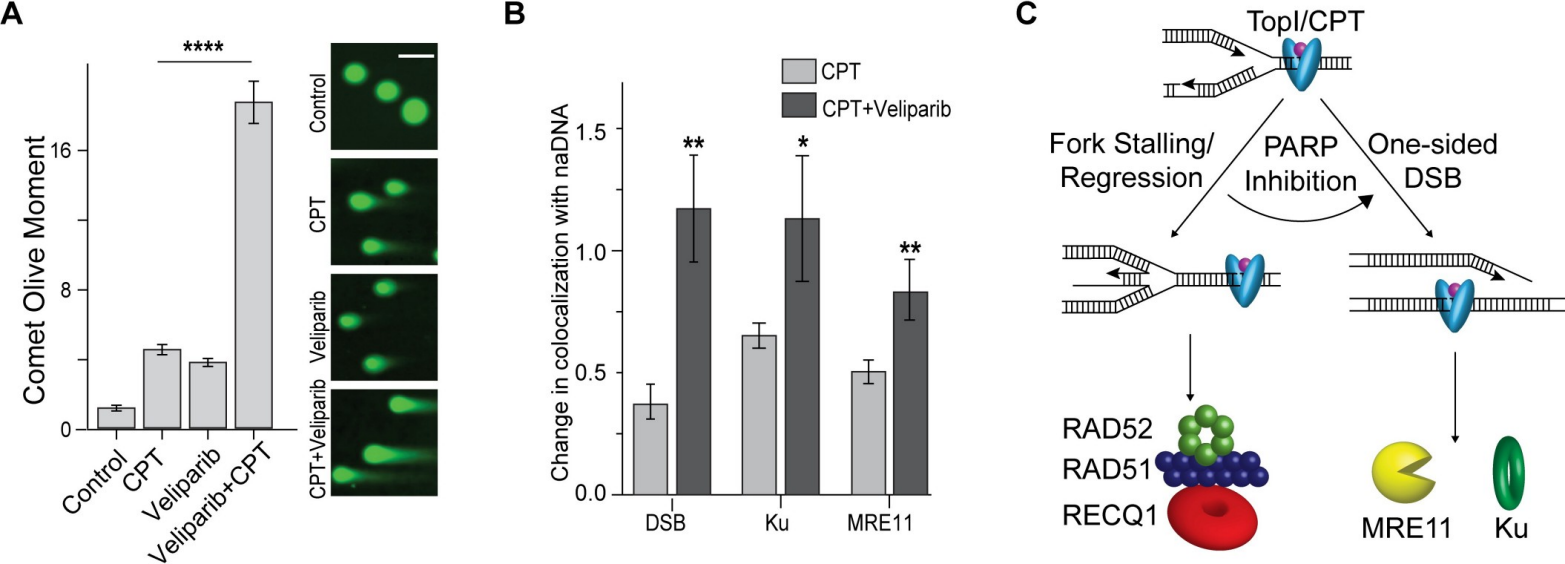
PARP inhibition converts RAD52/RAD51 protected RFs into seDSBs. (A) Comet assays of cells damaged with CPT, Veliparib or both demonstrating synergistic DSB induction upon TopI-induced RF stress combined with PARP inhibition. Representative cells shown. (B) Quantification of DSB (TUNEL), Ku and MRE11 association with naDNA upon PARP inhibition using a combined Veliparib/CPT treatment. (C) A model of the two predominant stress pathways induced by TopsIcc: RF stalling/regression, which is protected and repaired quickly and colocalizes with RAD51 and RAD52, and osDSBs, which colocalize with Ku and MRE11 and are repaired via HR. Representative comet assayed cells shown, scale bars represent 15 μm. Complete N values available in S1 and S2 Tables, number of replicates >3, number of cells imaged typically 20–60, number of individual foci manually scored typically 50–70. All graphs show mean ± s.e.m. Student’s t-test results shown for comparison with control levels in (A) and with CPT-only (B): ns depicts p>0.05, * depicts p<0.05, ** depicts p<0.01, **** depicts p<0.0001.

To test this hypothesis, we pre-treated cells with the PARP1/2 inhibitor Veliparib for 24 hours before addition of CPT. We used comet assays to determine the level of DSB induction to assess the degree of RF stalling/regression bypass resulting in increased seDSBs in the Veliparib+CPT-treated cells. While both these drugs generated increased levels of DSB induction individually, a synergistic effect was detected upon combination, showing that Veliparib was indeed causing CPT-induced replication stress to result in seDSBs (Fig 5A). This further confirmed the existence of a substantial number of stalled but unbroken RFs in CPT-treated cells. We also assessed Veliparib+CPT-induced DSB induction using SR and found increased levels of DSBs (via TUNEL), MRE11, and Ku association with naDNA foci (Fig 5B).

## Discussion

In this study we described powerful single-molecule localization-based assays for differentiating and analyzing distinct RF repair pathways. Using the enhanced spatial resolution and single molecule sensitivity achieved in these assays, we have been able to examine the organization of repair proteins within repair foci consisting of individual seDSBs or stalled/regressed RFs at a comparatively low level of damage induction. This enabled direct observation of specific, mutually exclusive first responder proteins: RAD51, RAD52, and RECQ1 at stalled forks, and MRE11 and Ku at seDSBs (Fig 5C). Together, these data constitute a direct visualization of closely linked damage motifs *in vivo* revealing hitherto unknown aspects of the specific roles of response proteins.

The two canonical DSB repair pathways, NHEJ and HR, are both active in the repair of chromosomal breaks during S phase [73]. The cellular abundance of Ku in human cells and its remarkably high affinity for a variety of DSB lesions results in its immediate recruitment to break sites [46]. Despite this, existing models of DSB repair in cells depict two exclusive pathways, each of which proceeds via a set of sequential steps. The data presented here demonstrate the existence of a more refined repair process in which Ku initially loads onto seDSBs generated at collapsed RFs despite their eventual repair via HR. Moreover, we show that MRE11 loads contemporaneously with Ku association, but in a distal position (Fig 3). This contradicts models in which MRE11 initially associates much closer to the DSB and progresses towards the break, suggesting not only that resection is initiated away from the DSB site but that MRE11 remains distal to the break and does not seem to simply execute 5’ to 3’ nucleolytic degradation as previously thought [16,22]. Instead our findings are in agreement with recent *in vitro* studies that showed MRE11 conducting long-range one-dimensional diffusion along dsDNA associated with a DSB [74]. These observations highlight the fact that all cellular DSBs are first bound by Ku, an event that is then followed by repair either via NHEJ or removal of Ku and progression of HR.

A noteworthy strength of our analysis rests on our ability to distinguish specific DNA damage response variability associated with different fork repair processes [17,75,76]. This led us to the observation that a subset of RFs colocalized with the HR proteins RAD51 and RAD52 immediately following CPT damage, but did not colocalize with TUNEL signal, Ku or MRE11 (Fig 4). These data are consistent with the notion that these forks do not form seDSBs. This distinctive DDR signature was also observed at forks subjected to replication stress via HU treatment, which specifically causes RF stalling and regression, whereas Ku and MRE11 were not detected in HU-treated cells (Fig 3A and 3B). Accordingly, we conclude that while low grade CPT treatment results in seDSB formation, it also yields unbroken but arrested RFs that require RAD51/RAD52 to mediate fork protection and regression processes [6]. This conclusion was further strengthened by detection of RECQ1, a helicase known to process regressed RFs (Fig 2E). Our observation of a lack of Ku, MRE11 and RPA at regressed RFs contrasts studies by others which made use of other cell and damage models and reported association of these proteins with stalled RFs, as well as more extensive resection [7,25,42]. This includes the recent characterization of a two-step progression of regressed fork resection regulated by Ku recruitment and later MRN removal of Ku [77]. We emphasize that our observations are likely specific to mammalian cells and transient, low-level stress within an endogenous context. This may allow regressed RFs to repair with less resection than has been observed in yeast studies, in cases of higher levels of damage, and in cells depleted of key protective proteins [25,42,77]. Furthermore, we highlight that our imaging assays, complementarily with other biochemical approaches, will likely prove useful in characterizing different regressed RF repair pathways and the conditions under which they operate.

Importantly, we showed that the activity of RAD51/RAD52 fork protection is PARP dependent, with combined Veliparib+CPT treatment resulting in conversion of regressed forks into seDSBs (Fig 5) [6]. This result further shows that endogenous stress caused by transcriptional activity and topographic remodeling ahead of RFs results in both stalled/regressed and broken RFs, and that these are repaired by distinct pathways, each making use of canonical HR proteins.

The novel combination of discriminatory labeling and analytical approaches with state-of-the-art super resolution imaging detailed here has enabled visualization and characterization of key endogenous damage species and subsequent cellular responses *in vivo*. We unequivocally demonstrate the coexistence of both arrested and broken RFs in cells under mild replication stress conditions. We further show recruitment of RECQ1, RAD51 and RAD52 to arrested RFs allowing for expeditious repair, and the loading of Ku and MRE11 specifically onto seDSBs, which are more slowly repaired via HR. The temporal and structural insights derived highlight the capabilities of the assays we have developed which we hope will be widely applied to complement other techniques in elucidating mechanisms of DNA damage and repair. Taken together, our observations offer new insights into the roles of various DNA damage species and the response pathways associated with the pathogenesis of cancer, autoimmune diseases, and a range of other genetic disorders.

## Material & methods

### Cell synchronization and drug treatment

Female human bone osteosarcoma U-2 OS cells (ATCC HTB-96) were routinely cultured in McCoy’s 5A (Modified) medium (ThermoFisher 16600) with 10% FBS (Gemini Bio. 100–106) and 100 U/mL Penicillin-Streptomycin (ThermoFisher 15140) at 37°C and 5% CO_2_.

Cells were seeded on No 1.5 glass coverslips and allowed to adhere in complete medium for 18–24 hours before being switched to FBS free medium for a further 48–72 hours in order to synchronize cells in G0/G1 phase [78]. Cells were subsequently released in complete medium for a further 16 hours to produce an early-mid S phase cell population. To induce RF stress and seDSB generation cells were treated with 100 nM CPT (Abcam 120115) [5] alongside pulse labeling with 10 μM EdU, a thymidine analogue, for one hour so that it would be incorporated into naDNA during replication (Click-iT kit, ThermoFisher C10340) [31]. Cells were fixed immediately following damage or released back into drug-free complete medium for 1–12 hours before later fixation. This allowed for spatiotemporal mapping of slower HR processes. Control cells were treated with 0.1% DMSO in place of CPT.

To further perturb and probe the HR process of DSBs, U2OS cells were treated with 100 μM of the PARP 1/2 inhibitor Veliparib (Santa Cruz, 202901) for 24 hours prior to recovery from CPT treatment [79]. To do this, cells seeded on coverslips were administered Veliparib during the final seven hours of serum starvation as well as during release in complete medium and in the CPT/EdU medium. Hydroxyurea experiments were conducted for 1–4 hours at a final concentration of 1 mM, whereas Neocarzinostatin was applied for 1 hour at 200 ng/ml.

### Extraction and Fixation

For clear visualization of chromatin and chromatin-bound nuclear fraction of cells, optimization of fixation and immunolabeling protocols was of paramount importance[80]. In particular, the methods presented here were specifically optimized to achieve a high degree of soluble component extraction while maintaining the ultrastructure of the nucleus and are in good agreement with similar protocols [46]. Pre-extraction of the cells was achieved using room temperature CSK buffer (10 mM Hepes, 300 mM Sucrose, 100 mM NaCl, 3 mM MgCl_2_, and 0.5% Triton X-100, pH = 7.4) for 2–3 minutes with gentle agitation. This was a key step as it removed the majority of the unbound fraction of HR proteins, which decreased the density of proteins within the nucleus and increased the proportion of detected proteins that were directly involved in HR (ie. associated with naDNA at DSB sites). This step also removed much of the cytosolic component, which minimized non-specific binding sites and sources of background and auto-fluorescence. After extraction, cells were fixed in paraformaldehyde (3.7% from 32% EM grade, Electron Microscopy Sciences, 15714) and glutaraldehyde (0.3% from 70% EM grade, Sigma-Aldrich, G7776) in PBS for 15 minutes. The cells were then washed three times with PBS and, if required, stored overnight at 4°C. For fluorescent tagging of the pulse labeled nascent DNA, the copper catalyzed ‘Click’ reaction was used as described in the Click-iT (ThermoFisher, C10640) protocol in conjunction with Alexa Fluor 647 [31]. The cells were blocked with blocking buffer (2% glycine, 2% BSA, 0.2% gelatin, and 50 mM NH_4_Cl in PBS) for 1 hour at room temperature (RT) or overnight at 4°C prior to further staining.

### TUNEL and immunofluorescence labeling

To visualize DSBs, the Click-iT TUNEL kit (ThermoFisher, C10247) was combined with ChromaTide Alexa Fluor 568-5-dUTP (ThermoFisher, C11399) and a modified protocol standardized against established ‘Click’ and Digoxigenin (digoxigenin-11-dUTP: Sigma, 11093088910, anti-digoxigenin: Novus, 31191) protocols [36]. The optimized protocol made use of conjugated AF568-5-dUTP (ThermoFisher, C11399) to label DSBs in conjunction with AF647 ‘Click’ labeled naDNA demonstrating high specificity of TUNEL labels as shown by high levels of overlap with naDNA foci in damaged cells. Following fixation and permeabilization, a TdT reaction mixture containing 94 μl TdT reaction buffer and 4 μl TdT was made up to a final concentration of 10 μM deoxyribose nucleotide triphosphates (dNTPs, ThermoFisher, R0191) and 50 μM AF568-5-dUTP. This was applied to a coverslip and allowed to react for 12 hours at 37°C and high humidity. In our hands we found that higher concentrations of dNTPs or shorter incubation times lowered that total number of TUNEL signals. We concluded that this was due to the much slower incorporation rate of the bulky AF568-5-dUTP compared to the natural dNTPs leading to some DSBs being detected and added to by the TdT, but only with dNTPs which were not fluorescently detectable. Samples were washed three times in PBS and blocked for a further 30 minutes with blocking buffer. Click reactions to detect EdU and antibody labelling was carried out after the TUNEL labelling.

Antibody labeling of all other proteins was achieved via a combination of direct and indirect labeling with Alexa Fluor 488 and 568 fluorophore labeled antibodies that have previously been validated by as and others for IF experiments. For a complete list, including validating references see S3 Table.

### Super resolution imaging

For SR imaging, coverslips were stored for up to one week at 4°C before being mounted onto a microscope microfluidics chamber just prior to imaging. SR imaging buffer comprising an oxygen scavenging system (1 mg/mL glucose oxidase (SigmaAldrich, G2133), 0.02 mg/mL catalase (SigmaAldrich, C3155), and 10% glucose (SigmaAldrich, G8270)) and 100 mM mercaptoethylamine (Fisher Scientific, BP2664100) in PBS was then prepared fresh and added to the imaging chamber.

A custom-built SR microscope based on a Leica DMI 3000 inverted microscope was used to acquire all the raw data as has been described previously [31]. Briefly, 473 nm (Opto Engine LLC, MBL-473-300 mW), 532 nm (OEM Laser Systems, MLL-III 200 mW) or 556 nm (UltraLasers, MGL-FN-556 200 mW) and 640 nm (OEM Laser Systems, MLL-III 150 mW) laser lines were combined using appropriate dichroics and focused onto the back aperture of a HCX PL APO 100X NA = 1.47 TIRF (Leica) objective via a multi-band dichroic (Chroma, zt405/488/532/640/730rpc, UF1C165837). The incident excitation beam could be translated laterally across the back of the objective to achieve a Highly Inclined and Laminated Optical (HILO) illumination configuration producing better signal-to-noise images. Fluorescence emission was collected through the same objective and dichroic and imaged on either an electron multiplying charge coupled device (EM-CCD) camera (Andor iXon+ 897) or scientific complementary metal-oxide semiconductor (sCMOS) camera (Photometrics, Prime 95B). Fluorescence signal from AF568 and AF647 could be collected simultaneously using a dual-band bandpass filter (Chroma, CY3/CY5, 59007m), and split into two channels on the EMCCD using a dichroic mirror (Semrock, FF660-Di02) in conjugation within a dual-view cube (Photometrics, DV2). AF488 signal was collected subsequently using a narrow single-band filter (Semrock, FF01-531/40). A 405 nm laser line (Applied Scientific Pro., SL-405 nm-300 mW) was introduced to enhance recovery of dark state fluorophores when required. Alternatively, channels were imaged sequentially. S phase nuclei were identified by positive AF647 naDNA signal and 2000 frames at 33 Hz were acquired for each color. Accumulation of more frames increased total signal only marginally (10–20%) and did not affect the overall analysis because immuno-, TUNEL, and naDNA labelling inherently involves multiple fluorophores targeted to the same underlying feature, and because complex structural determinations were not useful to the study. Representative images showing the blinking density and photobleaching can be seen in S6 Fig. Fitting uncertainty was determined based on photon number, PSF approximation, and the camera noise to be <10 nm in the red and blue channels, and <20 nm in the green channel. Mapping errors based on transformation of bead images and were found to be ~10 nm for both mapping of the blue channel to the red channel and the green channel to the red channel.

A polynomial morph-type mapping algorithm achieved offset and chromatic correctional mapping of the three channels. To generate the correctional map, each day, prior to imaging, diffraction-limited images of spatially separated broad-range emitting fluorescent beads were acquired across all three colors (Tetraspecks, 100 nm, Life Technologies, T7279). The precise localizations of the beads were obtained by independently fitting their diffraction-limited Point Spread Functions (PSF) with Gaussian functions. The three-color sub-diffraction localizations could then be matched and an elastic mapping matrix generated based on the polynomial morph-type mapping function using an IDL (Exelis Visual Information Solutions) custom mapping script. Three-color raw image stacks of cellular images were then corrected before SR analysis.

### Super resolution image rendering and analysis

To generate a table of molecular localizations and render SR images for colocalization analysis, the three acquired colors were processed independently using the ImageJ [81] plugin QuickPALM [82] with point spread function fitting constrained to spots with full-width half-maximum of 4–5 pixels (~264–535 nm) and signal-to-noise ratios better than 3. Images were rendered using 20 nm pixels and recombined to generate three-color micrographs over which masks were manually drawn identifying the nucleus based on the naDNA signal.

To determine the degree of colcoalization, Monte Carlo simulations depicting random distribution of the clusters relative to each other were used as a normalization approach. To achieve this, a random simulation was generated for each experimental image by creating grayscale images of two colors at a time from the image of a nucleus (ie. color-1 and color-2). The two greyscale images were then segmented by automatically applying an Otsu thresholding algorithm to the gray-scale pixels that belonged to the masked nuclear ROI. After cluster segmentation, a random redistribution of one of the colors was simulated by redrawing all of the clusters that belonged to the color-2 experimental image over the static color-1 image. From this ‘randomized’ simulation, both the number and the area of overlap between the two colors were determined.

Using this approach, 20 simulations of random rearrangements were generated for each nucleus in a pairwise fashion, examining Red/Green, Red/Blue, and Green/Blue overlap. The total number or area of overlaps detected in each nucleus (typically 15–100) could then be normalized to the determined random level of overlap expected by dividing the number or area of real overlaps by the average number or area of overlaps in the same simulated nucleus. An average normalized colocalization factor was thus calculated for each pairwise interaction between proteins, seDSB, ssDNA, and naDNA (N typically 30–50 total cells, each comprising 100s of foci, all N values available in S1 Table). A colocalization factor of 1 indicated completely random colocalization, while higher factors indicated association and interaction. Colocalization factors were also calculated for undamaged cells to establish control levels. Number of overlaps was used for TUNEL, TopI, Ku, MRE11, and BRCA1 whereas area of overlap was used for γH2AX, RPA, RAD51 and RAD52 due to the expected accumulation of these proteins over multiple time points. In general the trends observed in number of overlaps were comparable to those observed in area but were more striking in the latter case for accumulating proteins. Change in colocalization was further normalized against the degree of overlap detected in undamaged control cells fixed immediately following EdU pulse labeling. For this reason several overlaps show a negative change due to a decrease in protein association when the averaged damage foci is compared to the averaged replicating fork foci. For further explanation see [31,48].

To assess the prevalence of colocalization, dependence or exclusionary relationships between proteins at DSBs, all foci within a nucleus containing both naDNA and at least one of the two proteins stained were identified and quantified to determine the percentage of foci with colocalized proteins, as opposed to those positive only for one or the other. The average proportions were calculated and depicted in cumulative bar graphs.

To assess the intrafoci organization of proteins at DSBs, the distance between centers-of-mass of colocalized proteins at naDNA was determined. These distances were used to generate a histogram, which could be approximated with a single or double Gaussian. To standardize intrafoci two-color distances descriptive of closely associated proteins, RPA was double labeled with AF568 and AF488 and intrafoci distance histograms generated. In both cases, these histograms could be approximated with a Gaussian centered at an intrafoci distance of 135 nm with a width at half height of 70–80 nm. In cases where protein intrafoci distance histograms could similarly be fit with a Gaussian centered with the range 135 ± 37.5 nm, the relationship was considered intimate. In contrast, intrafoci distances which were approximated with a single Gaussian centered further than 180 nm described proteins occupying the same DSB but spatially separated. Histograms not easily described by a single Gaussian were well approximated by fitting one Gaussian at 135 nm distance and 75 nm full width at half height with free fitting of the total area of the Gaussian, and then free fitting a second Gaussian to describe the remaining intrafoci distances. Fitting two Gaussians described arrangements where in some foci the proteins were closely associated while in others they were spatially separated. To further visualize the spread of intrafoci distances observed, the fitted Gaussians were extrapolated into 3D contoured heat maps by fitting a second Gaussian perpendicular to the 2D Gaussian distance/intensity coordinates with the area of the perpendicular Gaussian proportional to the intensity value of the fitted Gaussians. These contoured heatmaps, shown throughout in blue, describe the likelihood of particular intrafoci distances being observed for different protein combinations at naDNA.

All experiments were carried out in duplicate or more with repeats performed to generate approximately normal data distributions with N sizes not predetermined. Unequal variances, particularly across temporal series, are expected. Manual ROI selection of nuclei for all quantification, including data assessment for pairwise interdependency assessment and intrafoci association distribution was carried out blinded to the drug condition, protein species and time point being quantified. The representative images of cell nuclei and foci shown throughout were rendered at 10 nm, smoothed, dilated, and brightened to make them easily assessable after resizing for inclusion in figures. Importantly, all quantitative analysis used here was applied to images that had not been manually processed, removing any user bias. For examples of SR data processing and rendering for both analysis and user-friendly presentation within the paper see S6 Fig.

### Comet assays

A neutral comet assay was used to specifically assess the amount of DNA DSBs in cells treated with Veliparib, CPT, or both. In individual wells of a six-well plate, cells were incubated with 0.4 mL of Trypsin for 10 minutes. 1 mL of medium was added to the trypsin and the cells centrifuged at 1000 g for 5 minutes. The supernatant was removed, and the cell pellet was resuspended in 500 μL of PBS. The suspended cells were added to prewarmed low-melting agarose at 37°C (10 μL of cells to 90 μL of agarose). The mix was pipetted onto a CometSlide (4250, Trevigen) and spread equally across the slide before being allowed to set for 30 minutes at 4°C. Following this, the slides were submerged in cold Lysis Buffer (4250, Trevigen) for 30 minutes on a shaker, and then in cold Tris/Borate/EDTA buffer. Electrophoresis was run for 15 minutes at 21V in a Mini-Sub Cell GT electrophoresis tray (Bio-Rad). Subsequently, cells were fixed in 70% ethanol and allowed to dry overnight. DNA was stained with Cygreen (GEN-105, ENZO) for 30 minutes, and slides were imaged on an EVOS fluorescence microscope (AMG) with appropriate filters for GFP imaging. Images acquired were processed using Open Comet software and the olive moment for each group of cells was calculated [83].

### Statistics

Statistical analysis was carried out in OriginLab (8.5). All naDNA/protein overlap colocalization factors calculated for controls and CPT-treated cells were tested against control samples (Student's two sample t-test). Combined Veliparib and CPT treatment was compared with controls, and with CPT-only samples, again using t-tests where appropriate.

## Supporting information

S1 Fig 1 hour 100 nM CPT treatment does not cause significant changes to EdU signal. (A-B) Representative undamaged cells immunolabelled for naDNA (magenta), γH2AX (cyan) and MRE11 (yellow). (C-D) Representative 1 hour 100 nM CPT treated cells stained as in (a-b) showing no visible difference in EdU signal and some increase in damage markers, compared to control cells. (E-F) Representative 3 hour 10 μM CPT treated cells stained as in (a-b) showing a loss of EdU signal and increased number/size of damage foci, compared to control cells. Scale bar = 10 μm
(TIF)

S2 Fig Individual repair foci comprise an individual seDSB and the 3D organization can be interpreted using 2D multicolor super resolution images. (A) To confirm the individual nature of single RFs within single foci we prepared cells in mid-S phase pulse labeled with EdU and immunlabeled for PCNA (PC10, Abcam) and MCM (EP2863Y, Abcam). (B-C) Elongated replicons were the prevalent species throughout the cell and showed the expected sequential nature of MCM-PCNA-naDNA confirming the RF was progressed as a single entity and not in a 'factory'. In conjunction with our regular detection of only single protein foci for any one naDNA we therefore concluded that we could assess naDNA foci as typically containing no, or one, damaged RF. (D-E) Cells pulse labeled with EdU were imaged in 3D to assess the typical axial depth of images contained using our highly inclined laminated optical sheet 2D acquisition setup. By moving the focus axially from a low focal plane (starting at 0 μm which we judged to be near the bottom of the nucleus) to two higher planes (+1.5 and +3.0 μm) and then resolving the EdU distribution in 3D we demonstrate that with HiLo illumination was are only sampling a ~1 μm thick slice of the cell, excluding interference from out of plane EdU. A representative cell is shown in 3D in the xy plane (D) and the xz plane (E, upper) with each of the three measurement planes depicted in a different color. E, lower, shows a single 3D projection in xz space demonstrating that the majority of detected molecules exist ±500 nm from the imaging plane. (F-H) Costaining of EdU, BRCA2, and RAD51 8 hours after CPT damage resulted in the characteristic 3D images of small repair foci showing all three colors. While different perspectives offered interesting insights into the intrafoci arrangement, colocalization was visible from all angles. (I-K) Quantification of the axial position of detected localizations in z-space for red, green, and blue labeled samples show axial sampling depths of 344 nm in blue, 422 nm in green, and 557 nm in red due to the chromatic differences in the emissions.
(TIF)

S3 Fig Three-tiered analytical approach for determining the overall kinetics of HR, the colocalization of proteins at single foci, and the internal organization of these proteins. Related to Figs 1–4. (A) A description, example, and principal characteristics elucidated by quantification of protein overlap with naDNA. Automated thresholding of SR images enabled pulse-labeled single RF naDNA foci to be defined and examined for overlap with ssDNA, TUNEL signal (DSBs), and various protein localizations. The total number of overlaps per cell was found to sensitively describe the kinetics of proteins expected to interact with stressed RFs in low numbers, whereas the total area of overlaps better described proteins expected to accumulate, such as RAD51, BRCA2, and RPA. (i) The number or area of naDNA/protein overlap was normalized to the level of overlaps predicted using randomized Monte Carlo simulations to account for the dense nuclear environment, and to the level of overlap detected in undamaged cells. (B) If proteins were found to significantly colocalize with naDNA after damage, they were further assessed in a pairwise fashion to quantify of the extent of co-occupancy at naDNA foci versus foci positive for only one or the other protein. In this way, we could determine the predominance of (i) colocalization, (ii) exclusivity, whereby the presence of one protein excluded the second protein from associating, (iii) dependence, whereby the presence of one protein was dependent on the presence of the other, or (iv) the prevalence of one protein over another. (C) Finally, pairwise labeling of proteins that yielded high incidences of protein-protein colocalization with naDNA were examined to determine the internal organization of proteins within these single foci. To do this, the distance between the centers of mass of the fluorophore localization clusters attributed to each protein was measured. A histogram of the distances detected could then be transformed into a 3D protein-protein distribution map which depicted either a (i) proximal or (ii) distal relationship between the two proteins under examination. This allowed differentiation between proteins that are spatially close to each other and potentially interacting from those that are at the same repair foci but are spatially separated and not interacting.
(TIF)

S4 Fig Temporal spatiokinetics of Ku, MRE11, and RAD51 at seDSBs. (A) Quantification of colocalization of Ku with naDNA foci in control cells and 0 and 1 hour after CPT-treatment showing Ku association only at 0 hours. (B) Quantification of colocalization of MRE11 with naDNA foci in control cells and 0, 1, 2 and 4 hours after CPT-treatment showing MRE11 association 0–2 hours indicating ongoing resection. (C) Quantification of colocalization of RAD51 with naDNA foci in control cells and 1, 2, 4, 8, and 12 hours after CPT-treatment showing no RAD51 association at 1 hour, peak RAD51 association at 2–4 hours before dissipation. This indicates the kinetics of RAD51/ssDNA nucleoprotein filament formation and homology search. Complete N values available in S1 and S2 Tables. All graphs show mean ± s.e.m. Student’s t-test results shown for comparison with control levels: ns depicts p>0.05, * depicts p<0.05, ** depicts p<0.01, *** depicts p<0.001, **** depicts p<0.0001.
(TIF)

S5 Fig Colocalization of DSBs and RECQ1, and MRE11 and RAD51, in control cells. (A) Quantification of colocalization of DSBs (TUNEL) with RECQ1 in control cells and cells damaged using NCS or HU demonstrating no significant association. (B) Quantification of colocalization of MRE11 with RAD51 foci in control cells and in cells immediately following damage using CPT, NCS and HU. Complete N values available in S1 and S2 Tables. All graphs show mean ± s.e.m. Student’s t-test results shown for comparison with control levels: ns depicts p>0.05
(TIF)

S6 Fig Raw and processed SR data showing blinking and rendering quality. (Ai) First and (ii) last frames in a representative raw image stack taken using red excitation/channel settings. (Bi-iv) Four sequential frames taken from the raw image stack as in (A), representative of typical mid-movie blinking. (C) The raw QuickPALM output image, brightened as a 32 bit image. (D) The Averaged histograms ThunderSTORM output image. (E) A zoom section from (C). (F) A zoom section as in (C) showing the ThunderSTORM scatter plot output. (G) A zoom section as in (C) showing the ThunderSTORM histogram plot output. (H) A zoom section as in (C) from (D) showing the ThunderSTORM comparison. (Ii) The QuickPALM image processed using smoothing and binarizing algorithms for display purposes within the manuscript. Analysis was performed on raw outputs as shown in (D). (ii) The coinciding zoom area. All zoomed out images including (A-B) are of ~25 μm across fields of view. All zoomed in frames are 2 μm across.
(TIF)

S7 Fig **Whole cell images corresponding to Fig 2** (A) Control undamaged cell labelled for naDNA and DSBs (TUNEL). (B) Control undamaged cell labelled for naDNA and TopI. (C) Control undamaged cell labelled for naDNA and γH2AX. (D) Control undamaged cell labelled for naDNA and MRE11. (E) Control undamaged cell labelled for naDNA and Ku. (F) CPT-treated cell labelled for naDNA and Ku. (G) Control undamaged cell labelled for naDNA and RECQ1. (H) CPT-treated cell labelled for naDNA and RECQ1. Scale bars show 5 μm.
(TIF)

S8 Fig **Whole cell images corresponding to Fig 3A and 3B showing DSB (TUNEL) labelling** (A) Control undamaged cell labelled for Ku and DSBs (TUNEL). (B) NCS-treated cell labelled for Ku and DSBs (TUNEL). (C) HU-treated cell labelled for Ku and DSBs (TUNEL). (D) Control undamaged cell labelled for MRE11 and DSBs (TUNEL). (E) NCS-treated cell labelled for MRE11 and DSBs (TUNEL). (F) HU-treated cell labelled for MRE11 and DSBs (TUNEL). Scale bars show 5 μm.
(TIF)

S9 Fig Whole cell images corresponding to Fig 3C–3F (A) Control undamaged cell labelled for naDNA, TopI and DSBs (TUNEL). (B) CPT-treated cell labelled for naDNA, TopI and DSBs (TUNEL). (C) Control undamaged cell labelled for naDNA, Ku and DSBs (TUNEL). (D) CPT-treated cell labelled for naDNA, Ku and DSBs (TUNEL). (E) Control undamaged cell labelled for naDNA, MRE11 and DSBs (TUNEL). (F) CPT-treated cell labelled for naDNA, MRE11 and DSBs (TUNEL). (G) Control undamaged cell labelled for naDNA, MRE11 and Ku. (H) CPT-treated cell labelled for naDNA, MRE11 and Ku. Scale bars show 5 μm.
(TIF)

S10 Fig Whole cell images corresponding to Fig 4A–4G. (A) Control undamaged cell labelled for naDNA, RAD51 and RAD52. (B) CPT-treated cell labelled for naDNA, RAD51 and RAD52. (C) Control undamaged cell labelled for naDNA, RAD51 and DSBs (TUNEL). (D) CPT-treated cell labelled for naDNA, RAD51 and DSBs (TUNEL). (E) Control undamaged cell labelled for naDNA, Ku and RAD51. (F) CPT-treated cell labelled for naDNA, Ku and RAD51. (G) Control undamaged cell labelled for naDNA, MRE11 and RAD51. (H) CPT-treated cell labelled for naDNA, MRE11 and RAD51. (I) Control undamaged cell labelled for naDNA, RAD51 and RECQ1. (J) CPT-treated cell labelled for naDNA, RAD51 and RECQ1. Scale bars show 5 μm.
(TIF)

S1 Table N values for overlap analyses. (PDF)

S2 Table N values for intrafoci analyses of WT+CPT damaged cells. (PDF)

S3 Table Antibody List. (PDF)
