# Supplementary figures and images for: Super-resolution visualization of distinct stalled and broken replication fork structures
Source: PLoS Genet. 2020 Dec 28;16(12):e1009256. doi: 10.1371/journal.pgen.1009256 (PMC7793303; doi:10.1371/journal.pgen.1009256)

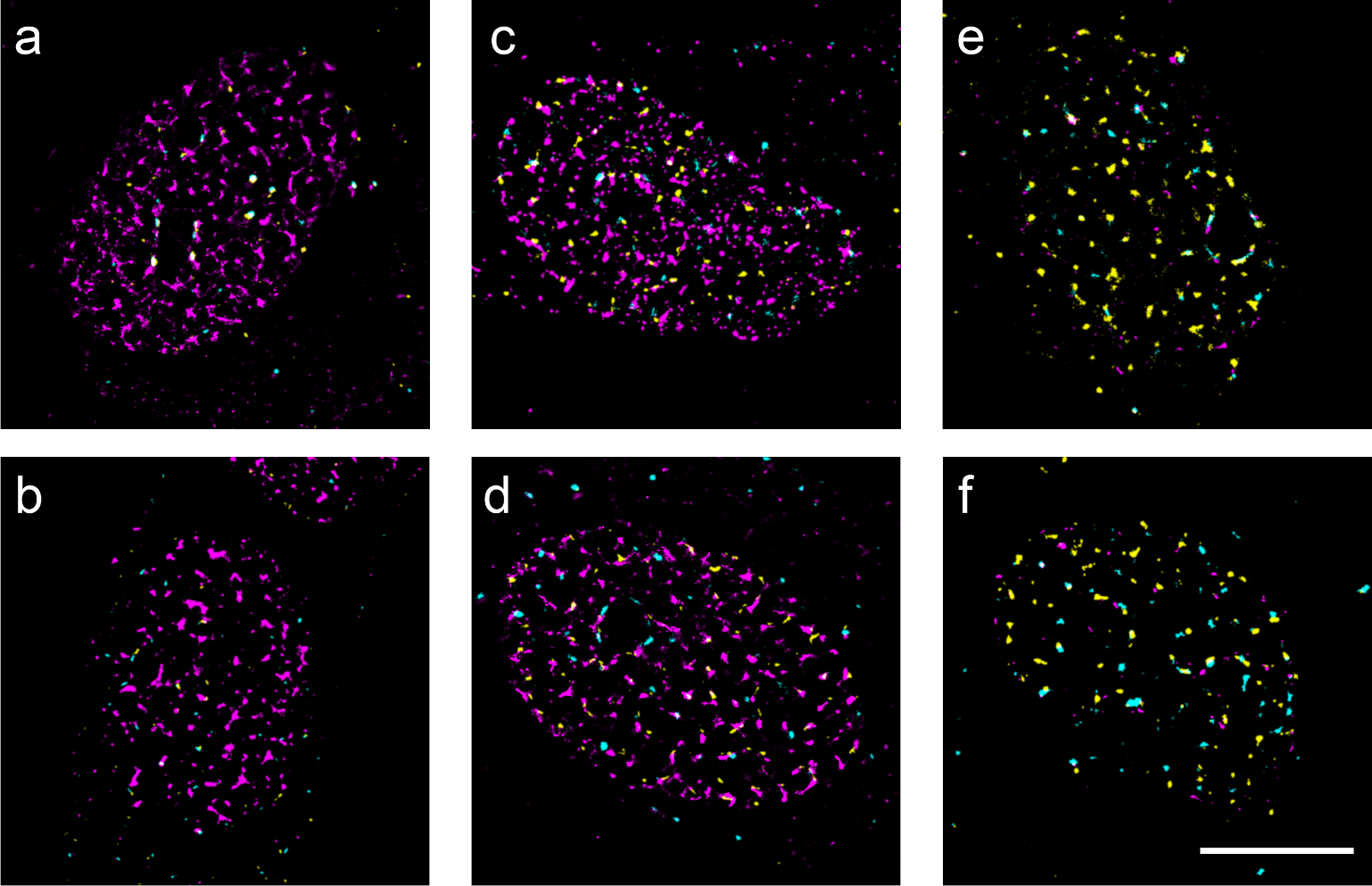

Supplement: S1 Fig — (A-B) Representative undamaged cells immunolabelled for naDNA (magenta), γH2AX (cyan) and MRE11 (yellow). (C-D) Representative 1 hour 100 nM CPT treated cells stained as in (a-b) showing no visible difference in EdU signal and some increase in damage markers, compared to control cells. (E-F) Representative 3 hour 10 μM CPT treated cells stained as in (a-b) showing a loss of EdU signal and increased number/size of damage foci, compared to control cells. Scale bar = 10 μm (TIF) [file pgen.1009256.s001.tif]

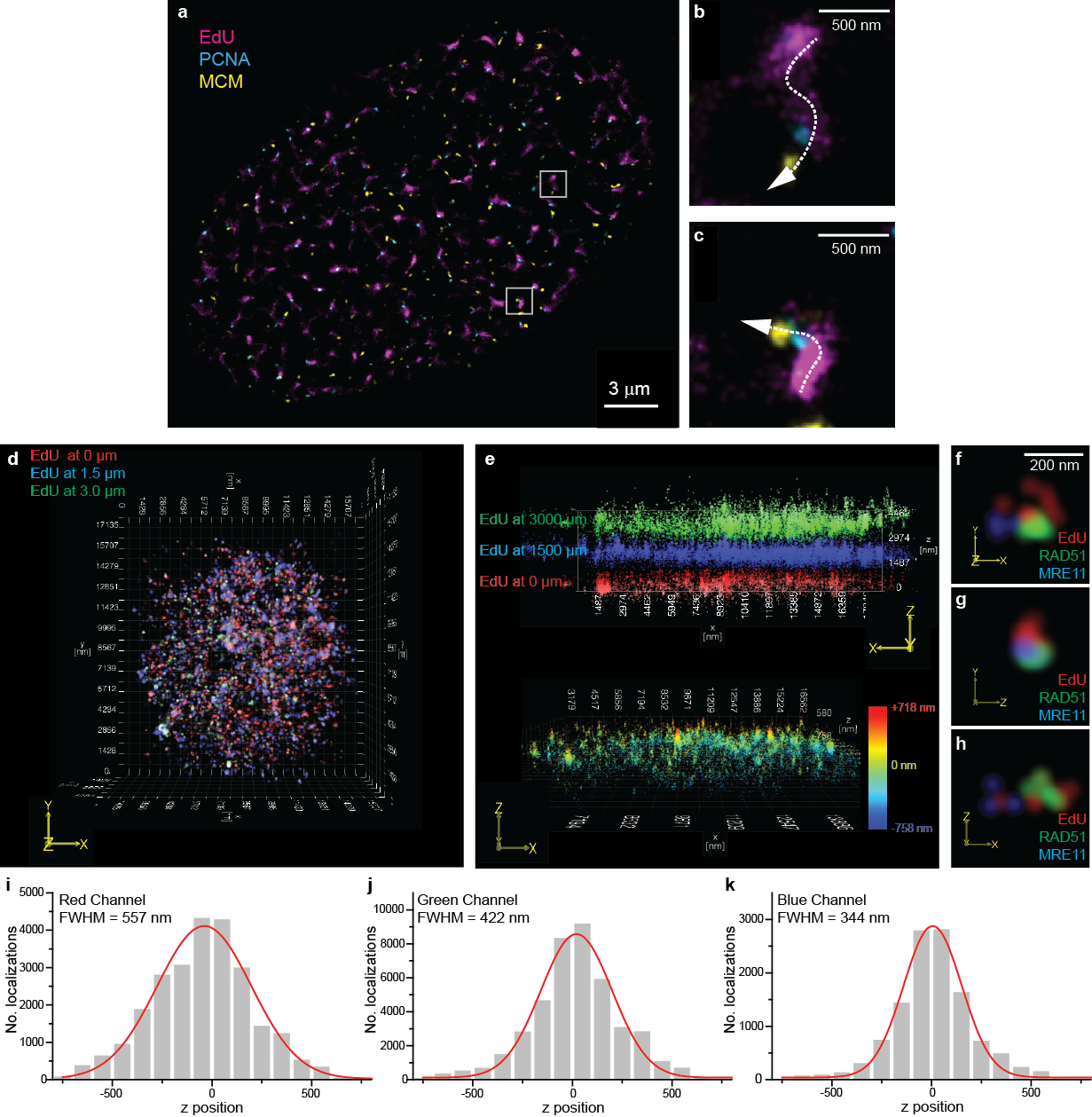

Supplement: S2 Fig — (A) To confirm the individual nature of single RFs within single foci we prepared cells in mid-S phase pulse labeled with EdU and immunlabeled for PCNA (PC10, Abcam) and MCM (EP2863Y, Abcam). (B-C) Elongated replicons were the prevalent species throughout the cell and showed the expected sequential nature of MCM-PCNA-naDNA confirming the RF was progressed as a single entity and not in a 'factory'. In conjunction with our regular detection of only single protein foci for any one naDNA we therefore concluded that we could assess naDNA foci as typically containing no, or one, damaged RF. (D-E) Cells pulse labeled with EdU were imaged in 3D to assess the typical axial depth of images contained using our highly inclined laminated optical sheet 2D acquisition setup. By moving the focus axially from a low focal plane (starting at 0 μm which we judged to be near the bottom of the nucleus) to two higher planes (+1.5 and +3.0 μm) and then resolving the EdU distribution in 3D we demonstrate that with HiLo illumination was are only sampling a ~1 μm thick slice of the cell, excluding interference from out of plane EdU. A representative cell is shown in 3D in the xy plane (D) and the xz plane (E, upper) with each of the three measurement planes depicted in a different color. E, lower, shows a single 3D projection in xz space demonstrating that the majority of detected molecules exist ±500 nm from the imaging plane. (F-H) Costaining of EdU, BRCA2, and RAD51 8 hours after CPT damage resulted in the characteristic 3D images of small repair foci showing all three colors. While different perspectives offered interesting insights into the intrafoci arrangement, colocalization was visible from all angles. (I-K) Quantification of the axial position of detected localizations in z-space for red, green, and blue labeled samples show axial sampling depths of 344 nm in blue, 422 nm in green, and 557 nm in red due to the chromatic differences in the emissions. (TIF) [file pgen.1009256.s002.tif]

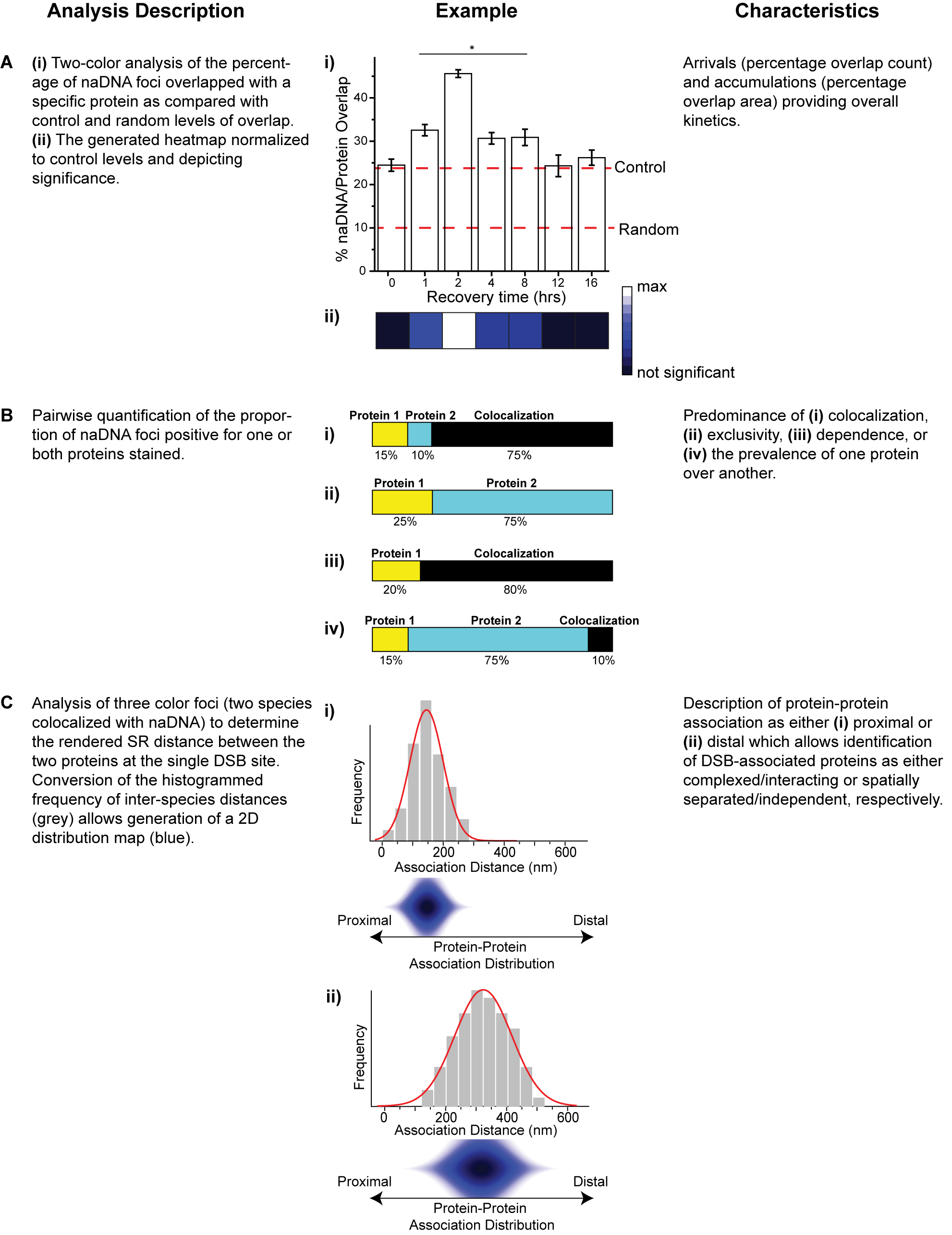

Supplement: S3 Fig — Related to Figs 1–4. (A) A description, example, and principal characteristics elucidated by quantification of protein overlap with naDNA. Automated thresholding of SR images enabled pulse-labeled single RF naDNA foci to be defined and examined for overlap with ssDNA, TUNEL signal (DSBs), and various protein localizations. The total number of overlaps per cell was found to sensitively describe the kinetics of proteins expected to interact with stressed RFs in low numbers, whereas the total area of overlaps better described proteins expected to accumulate, such as RAD51, BRCA2, and RPA. (i) The number or area of naDNA/protein overlap was normalized to the level of overlaps predicted using randomized Monte Carlo simulations to account for the dense nuclear environment, and to the level of overlap detected in undamaged cells. (B) If proteins were found to significantly colocalize with naDNA after damage, they were further assessed in a pairwise fashion to quantify of the extent of co-occupancy at naDNA foci versus foci positive for only one or the other protein. In this way, we could determine the predominance of (i) colocalization, (ii) exclusivity, whereby the presence of one protein excluded the second protein from associating, (iii) dependence, whereby the presence of one protein was dependent on the presence of the other, or (iv) the prevalence of one protein over another. (C) Finally, pairwise labeling of proteins that yielded high incidences of protein-protein colocalization with naDNA were examined to determine the internal organization of proteins within these single foci. To do this, the distance between the centers of mass of the fluorophore localization clusters attributed to each protein was measured. A histogram of the distances detected could then be transformed into a 3D protein-protein distribution map which depicted either a (i) proximal or (ii) distal relationship between the two proteins under examination. This allowed differentiation between protei [file pgen.1009256.s003.tif]

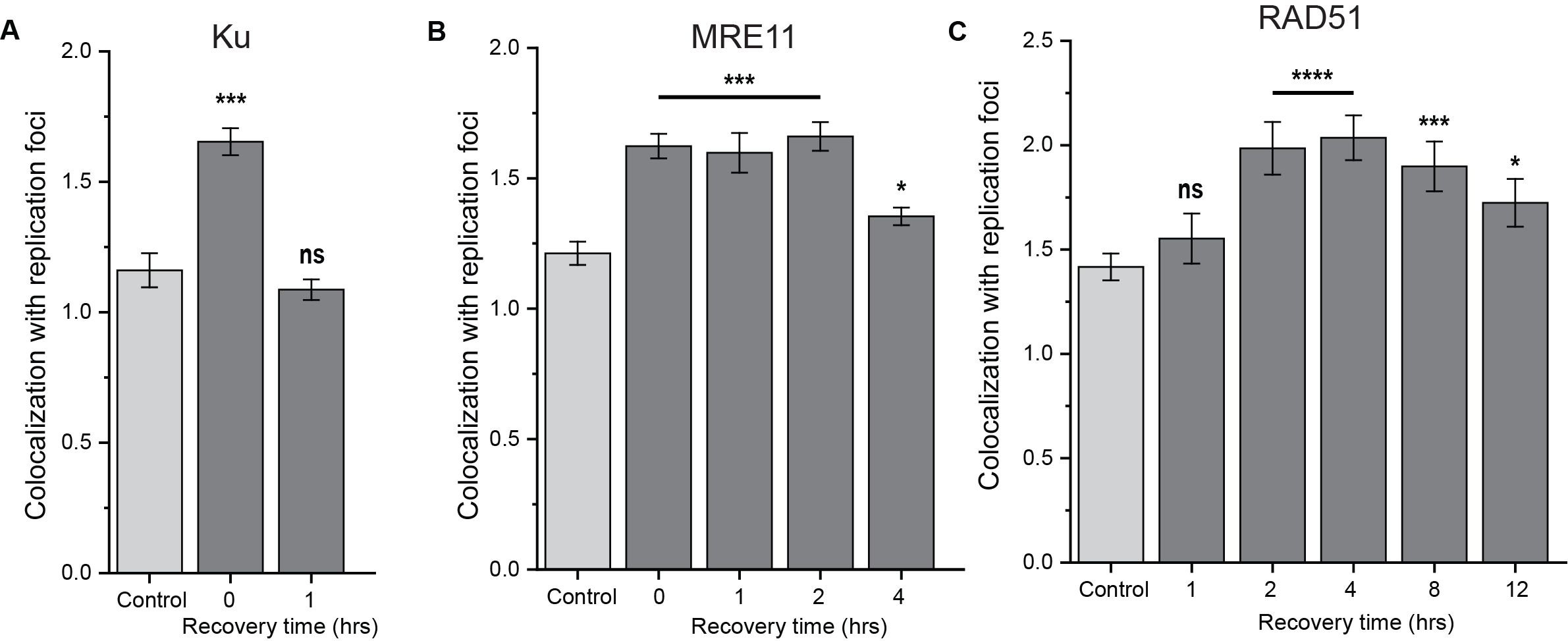

Supplement: S4 Fig — (A) Quantification of colocalization of Ku with naDNA foci in control cells and 0 and 1 hour after CPT-treatment showing Ku association only at 0 hours. (B) Quantification of colocalization of MRE11 with naDNA foci in control cells and 0, 1, 2 and 4 hours after CPT-treatment showing MRE11 association 0–2 hours indicating ongoing resection. (C) Quantification of colocalization of RAD51 with naDNA foci in control cells and 1, 2, 4, 8, and 12 hours after CPT-treatment showing no RAD51 association at 1 hour, peak RAD51 association at 2–4 hours before dissipation. This indicates the kinetics of RAD51/ssDNA nucleoprotein filament formation and homology search. Complete N values available in S1 and S2 Tables. All graphs show mean ± s.e.m. Student’s t-test results shown for comparison with control levels: ns depicts p>0.05, * depicts p<0.05, ** depicts p<0.01, *** depicts p<0.001, **** depicts p<0.0001. (TIF) [file pgen.1009256.s004.tif]

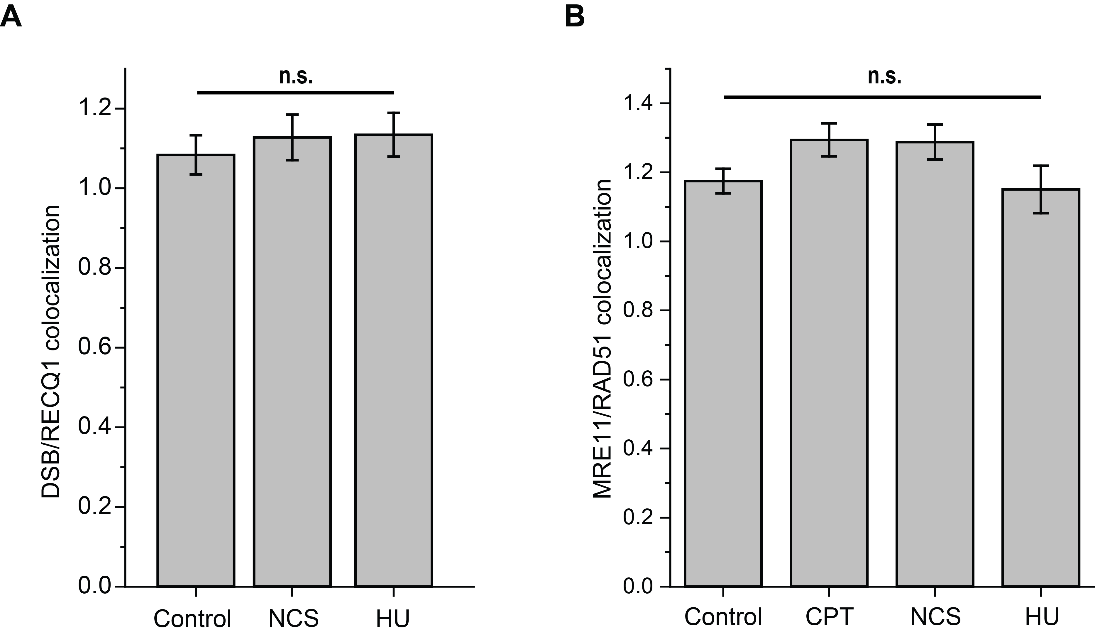

Supplement: S5 Fig — (A) Quantification of colocalization of DSBs (TUNEL) with RECQ1 in control cells and cells damaged using NCS or HU demonstrating no significant association. (B) Quantification of colocalization of MRE11 with RAD51 foci in control cells and in cells immediately following damage using CPT, NCS and HU. Complete N values available in S1 and S2 Tables. All graphs show mean ± s.e.m. Student’s t-test results shown for comparison with control levels: ns depicts p>0.05 (TIF) [file pgen.1009256.s005.tif]

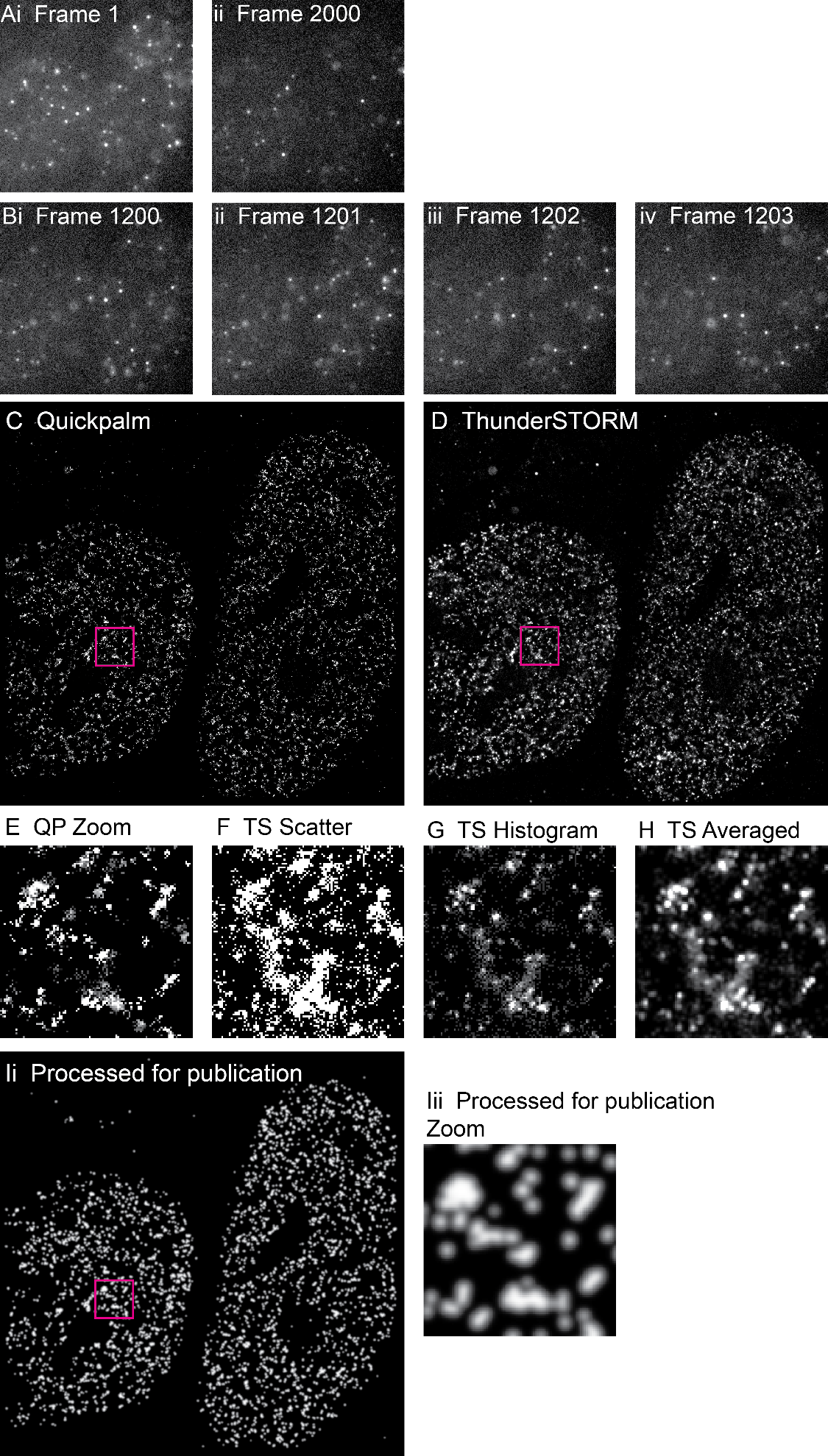

Supplement: S6 Fig — (Ai) First and (ii) last frames in a representative raw image stack taken using red excitation/channel settings. (Bi-iv) Four sequential frames taken from the raw image stack as in (A), representative of typical mid-movie blinking. (C) The raw QuickPALM output image, brightened as a 32 bit image. (D) The Averaged histograms ThunderSTORM output image. (E) A zoom section from (C). (F) A zoom section as in (C) showing the ThunderSTORM scatter plot output. (G) A zoom section as in (C) showing the ThunderSTORM histogram plot output. (H) A zoom section as in (C) from (D) showing the ThunderSTORM comparison. (Ii) The QuickPALM image processed using smoothing and binarizing algorithms for display purposes within the manuscript. Analysis was performed on raw outputs as shown in (D). (ii) The coinciding zoom area. All zoomed out images including (A-B) are of ~25 μm across fields of view. All zoomed in frames are 2 μm across. (TIF) [file pgen.1009256.s006.tif]

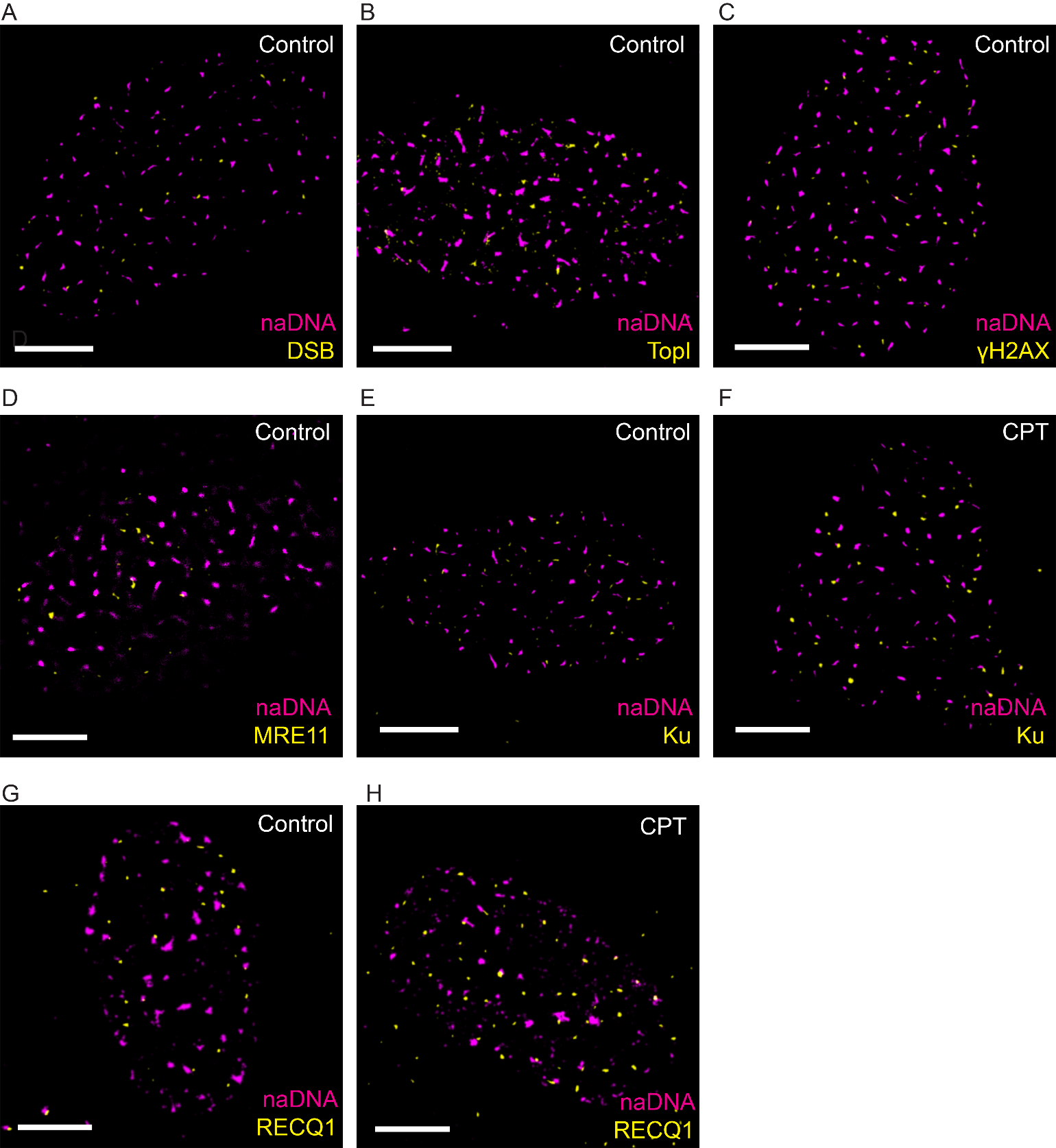

Supplement: S7 Fig — Whole cell images corresponding to Fig 2 (A) Control undamaged cell labelled for naDNA and DSBs (TUNEL). (B) Control undamaged cell labelled for naDNA and TopI. (C) Control undamaged cell labelled for naDNA and γH2AX. (D) Control undamaged cell labelled for naDNA and MRE11. (E) Control undamaged cell labelled for naDNA and Ku. (F) CPT-treated cell labelled for naDNA and Ku. (G) Control undamaged cell labelled for naDNA and RECQ1. (H) CPT-treated cell labelled for naDNA and RECQ1. Scale bars show 5 μm. (TIF) [file pgen.1009256.s007.tif]

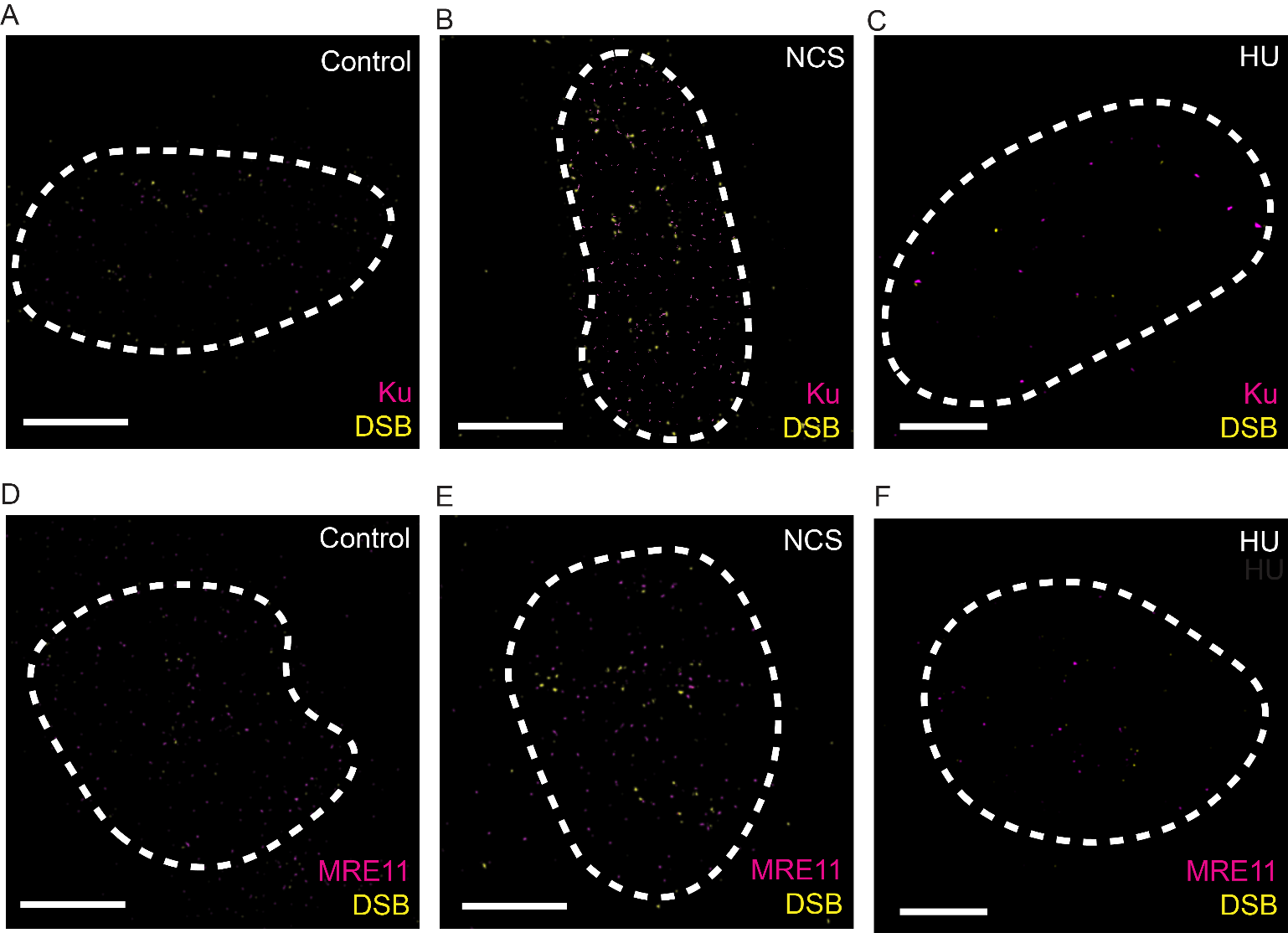

Supplement: S8 Fig — Whole cell images corresponding to Fig 3A and 3B showing DSB (TUNEL) labelling (A) Control undamaged cell labelled for Ku and DSBs (TUNEL). (B) NCS-treated cell labelled for Ku and DSBs (TUNEL). (C) HU-treated cell labelled for Ku and DSBs (TUNEL). (D) Control undamaged cell labelled for MRE11 and DSBs (TUNEL). (E) NCS-treated cell labelled for MRE11 and DSBs (TUNEL). (F) HU-treated cell labelled for MRE11 and DSBs (TUNEL). Scale bars show 5 μm. (TIF) [file pgen.1009256.s008.tif]

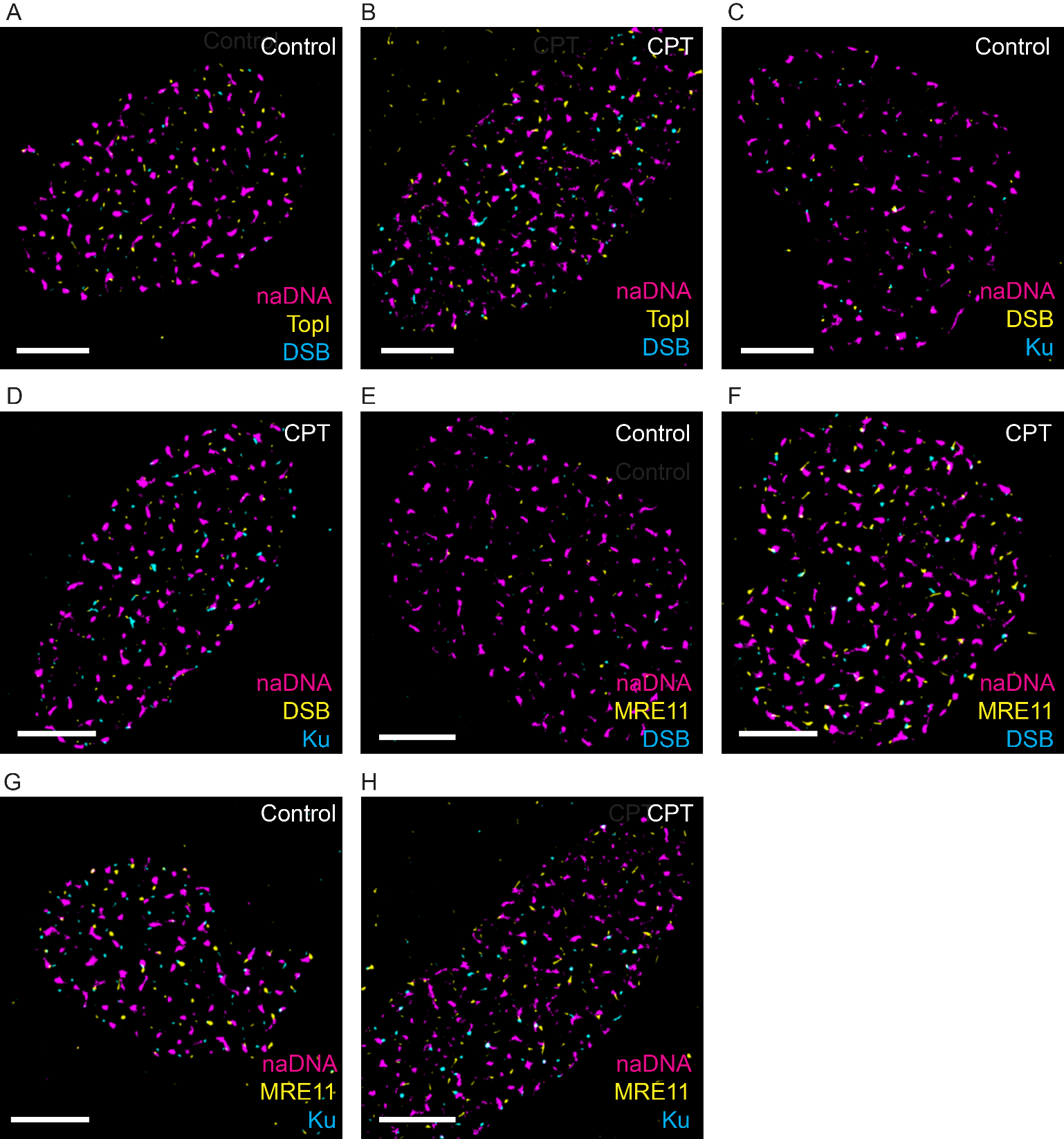

Supplement: S9 Fig — (B) CPT-treated cell labelled for naDNA, TopI and DSBs (TUNEL). (C) Control undamaged cell labelled for naDNA, Ku and DSBs (TUNEL). (D) CPT-treated cell labelled for naDNA, Ku and DSBs (TUNEL). (E) Control undamaged cell labelled for naDNA, MRE11 and DSBs (TUNEL). (F) CPT-treated cell labelled for naDNA, MRE11 and DSBs (TUNEL). (G) Control undamaged cell labelled for naDNA, MRE11 and Ku. (H) CPT-treated cell labelled for naDNA, MRE11 and Ku. Scale bars show 5 μm. (TIF) [file pgen.1009256.s009.tif]

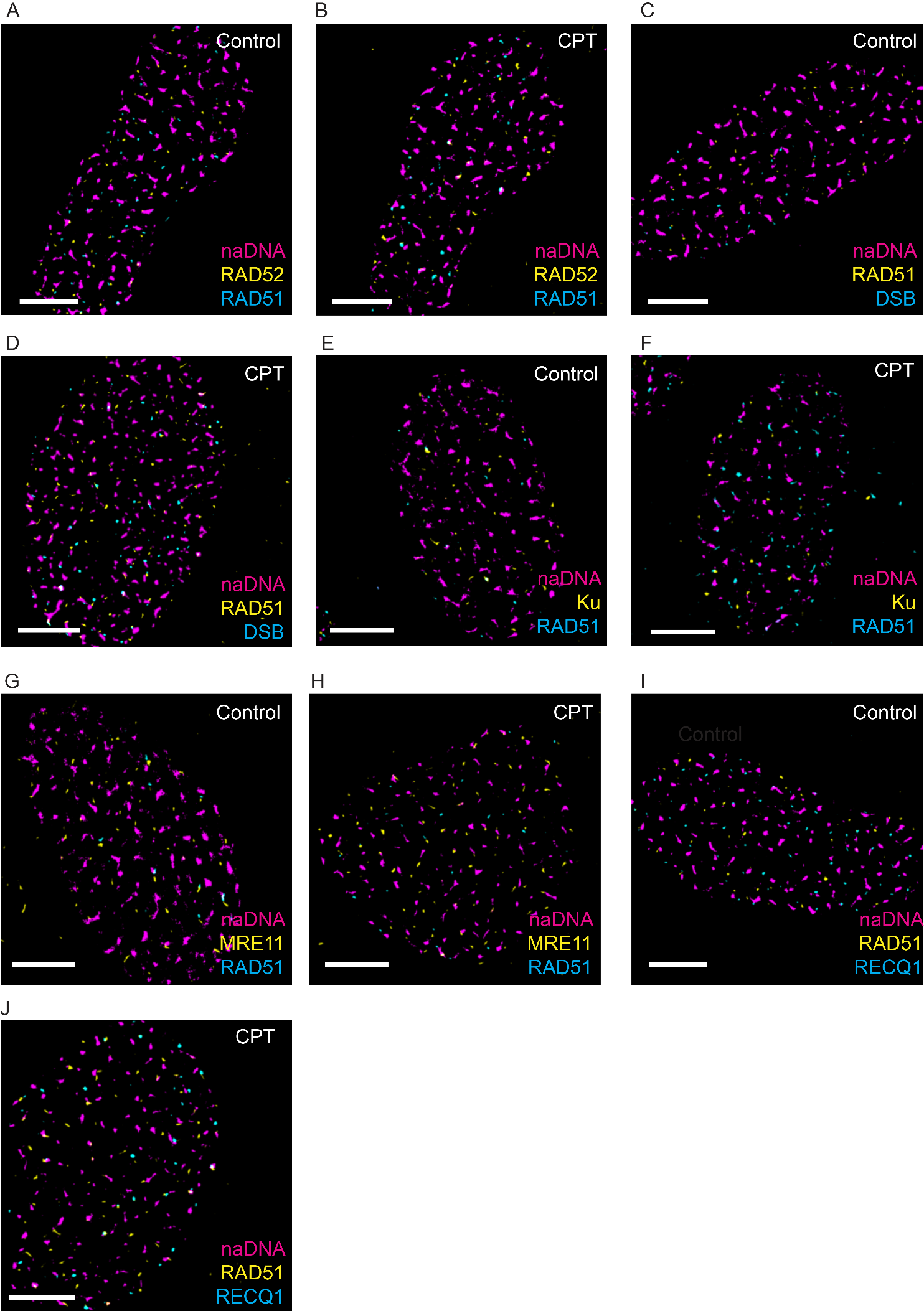

Supplement: S10 Fig — (A) Control undamaged cell labelled for naDNA, RAD51 and RAD52. (B) CPT-treated cell labelled for naDNA, RAD51 and RAD52. (C) Control undamaged cell labelled for naDNA, RAD51 and DSBs (TUNEL). (D) CPT-treated cell labelled for naDNA, RAD51 and DSBs (TUNEL). (E) Control undamaged cell labelled for naDNA, Ku and RAD51. (F) CPT-treated cell labelled for naDNA, Ku and RAD51. (G) Control undamaged cell labelled for naDNA, MRE11 and RAD51. (H) CPT-treated cell labelled for naDNA, MRE11 and RAD51. (I) Control undamaged cell labelled for naDNA, RAD51 and RECQ1. (J) CPT-treated cell labelled for naDNA, RAD51 and RECQ1. Scale bars show 5 μm. (TIF) [file pgen.1009256.s010.tif]
