# Supplementary material for: Super-resolution visualization of distinct stalled and broken replication fork structures
Source: PLoS Genet. 2020 Dec 28;16(12):e1009256. doi: 10.1371/journal.pgen.1009256 (PMC7793303; doi:10.1371/journal.pgen.1009256)
Supplement: S1 Table — (PDF) [file pgen.1009256.s011.pdf]

S1 Table: N values for overlap analyses.

Drug Condition	Time	Species 1	Species 2	N
Control	0	naDNA	yH2A.X	30
CPT Only	0	naDNA	yH2A.X	40
Control	0	naDNA	TUNEL	31
CPT Only (Fig 1C)	0	naDNA	TUNEL	82
CPT Only	0	naDNA	TUNEL	42
CPT +Veliparib	0	naDNA	TUNEL	62
Control	0	naDNA	Ku	19
CPT Only (Fig 1C)	0	naDNA	Ku	121
CPT Only	0	naDNA	Ku	103
CPT Only	1	naDNA	Ku	51
CPT+Veliparib	0	naDNA	Ku	42
Control	0	naDNA	MRE11	37
CPT Only (Fig 1C)	0	naDNA	MRE11	99
CPT Only	0	naDNA	MRE11	96
CPT Only	1	naDNA	MRE11	48
CPT Only	2	naDNA	MRE11	104
CPT Only	4	naDNA	MRE11	76
CPT+Veliparib	0	naDNA	MRE11	46
Control	0	naDNA	RAD52	18
CPT Only	0	naDNA	RAD52	24
CPT Only	1	naDNA	RAD52	13
HU	0	naDNA	RAD52	25
HU	1.5	naDNA	RAD52	31
Control	0	RAD51	RAD52	39
CPT Only	0	RAD51	RAD52	27
CPT Only	1	RAD51	RAD52	19

Drug Condition	Time	Species 1	Species 2	N
Control	0	naDNA	BRCA1	46
HU	0	naDNA	BRCA1	30
HU	1.5	naDNA	BRCA1	30
Control	0	naDNA	RAD51	67
CPT Only	0	naDNA	RAD51	57
CPT Only	1	naDNA	RAD51	49
CPT Only	2	naDNA	RAD51	50
CPT Only	4	naDNA	RAD51	47
CPT Only	8	naDNA	RAD51	53
CPT Only	12	naDNA	RAD51	42
HU	0	naDNA	RAD51	34
HU	1.5	naDNA	RAD51	39
Control	0	naDNA	RECQ1	56
CPT Only (Fig 1C)	0	naDNA	RECQ1	78
CPT Only	0	naDNA	RECQ1	49
Control	0	naDNA	RPA	63
HU	0	naDNA	RPA	34
HU	1.5	naDNA	RPA	39
Control	0	naDNA	TopI	24
CPT Only	0	naDNA	TopI	35
Control	0	DSB	RAD51	71
NCS	0	DSB	RAD51	64
Control	0	DSB	RAD52	72
NCS	0	DSB	RAD52	45

**Drug Condition**	**Time**	**Species 1**	**Species 2**	**N**
Control	0	DSB	Ku	61
NCS	0	DSB	Ku	72
HU	0	DSB	Ku	51
Control	0	DSB	MRE11	56
NCS	0	DSB	MRE11	36
HU	0	DSB	MRE11	70
