# Supplementary material for: Super-resolution visualization of distinct stalled and broken replication fork structures
Source: PLoS Genet. 2020 Dec 28;16(12):e1009256. doi: 10.1371/journal.pgen.1009256 (PMC7793303; doi:10.1371/journal.pgen.1009256)
Supplement: S2 Table — (PDF) [file pgen.1009256.s012.pdf]

S2 Table: N values for intrafoci analyses of WT+CPT damaged cells.

Time	Species 1	Species 2	N
0	MRE11	Ku	107
0	TopI	DSB	65
0	MRE11	DSB	51
0	Ku	DSB	53
0	RAD51	DSB	94
0	Ku	RAD51	101
0	MRE11	RAD51	76
0	RAD52	RAD51	58
0	Ku	RECQ1	145
0	RAD51	RECQ1	101
