# Supplementary material for: Super-resolution visualization of distinct stalled and broken replication fork structures
Source: PLoS Genet. 2020 Dec 28;16(12):e1009256. doi: 10.1371/journal.pgen.1009256 (PMC7793303; doi:10.1371/journal.pgen.1009256)
Supplement: S3 Table — (PDF) [file pgen.1009256.s013.pdf]

S3 Table: Antibody List.

Target	Species/Conjugate	Product Code	Manufacturer	Dilutions	Refs
BRCA1	mouse monoclonal AF488 conjugated	NB100-598AF488	Novus	1:250	*
Ku	mouse monoclonal	ms-286	ThermoFisher	1:1000/1:5000	1
MRE11	rabbit polyclonal	NB100-142	Novus	1:500/1:2000	2, 3
RAD51	rabbit monoclonal AF488 conjugated	AB196449	Abcam	1:250	*
RAD51	rabbit polyclonal	39194	Active Motif	1:500/1:1000	4
RAD51	mouse monoclonal	GTX70230	Genetex	1:500/1:2000	5
RAD52	rabbit polyclonal	SC8350	Santa Cruz	1:400/1:2000	6
RECQ1	rabbit polyclonal	Ab151501	Abcam	1:500/1:2000	*
RECQ1	mouse polyclonal	Ab89817	Abcam	1:200/1:1000	*
RPA	mouse monoclonal	AB2175	Abcam	1:500/1:2000	7, 8
RPA	rabbit monoclonal AF488 conjugated	AB199097	Abcam	1:300	9
TopI	rabbit polyclonal	AB3825	Abcam	1:500/1:2000	10
yH2A.X	rabbit polyclonal	NB100-384	Novus	1:2000/1:10000	11,12
yH2A.X	mouse monoclonal	05-636	EMD Millipore	1:2000/1:10000	13
	goat-anti-rabbit AF568	A11036	Invitrogen		
	goat-anti-rabbit AF488	A11034	Invitrogen		
	goat-anti-mouse AF568	A11031	Invitrogen		
	goat anti-mouse AF488	A11029	Invitrogen		

* denotes antibodies used which had not been used for IF applications in publications previously. To validate these antibodies they were double-stained alongside antibodies for the same target and found to have good colocalization.

1 Reid, D. A. *et al.* Organization and dynamics of the nonhomologous end-joining machinery during DNA double-strand break repair. *Proc. Natl. Acad. Sci. U. S. A.* **112**, E2575-E2584, doi:10.1073/pnas.1420115112 (2015).

2 Lee, K. Y. *et al.* MCM8-9 complex promotes resection of double-strand break ends by MRE11-RAD50-NBS1 complex. *Nature Communications* **6**, doi:10.1038/ncomms8744 (2015).

3 Gao, M. *et al.* Ago2 facilitates Rad51 recruitment and DNA double-strand break repair by homologous recombination. *Cell Research* **24**, 532-541, doi:10.1038/cr.2014.36 (2014).

4 Bennett, B. T. & Knight, K. L. Cellular localization of human Rad51C and regulation of ubiquitin-mediated proteolysis of Rad51. *J. Cell. Biochem.* **96**, 1095-1109, doi:10.1002/jcb.20640 (2005).

5 Pfaffle, H. N. *et al.* EGFR-Activating Mutations Correlate with a Fanconi Anemia-like Cellular Phenotype That Includes PARP Inhibitor Sensitivity. *Cancer Res.* **73**, 6254-6263, doi:10.1158/0008-5472.can-13-0044 (2013).

6 Wray, J., Liu, J. M., Nickoloff, J. A. & Shen, Z. Y. Distinct RAD51 associations with RAD52 and BCCIP in response to DNA damage and replication stress. *Cancer Res.* **68**, 2699-2707, doi:10.1158/0008-5472.can-07-6505 (2008).

7 Leung, J. W. *et al.* Nucleosome Acidic Patch Promotes RNF168-and RING1B/BMI1-Dependent H2AX and H2A Ubiquitination and DNA Damage Signaling. *Plos Genetics* **10**, doi:10.1371/journal.pgen.1004178 (2014).

8 Carvalho, S. *et al.* SETD2 is required for DNA double-strand break repair and activation of the p53-mediated checkpoint. *Elife* **3**, doi:10.7554/eLife.02482 (2014).

9 Toledo, L. I. *et al.* ATR Prohibits Replication Catastrophe by Preventing Global Exhaustion of RPA. *Cell* **155**, 1088-1103, doi:10.1016/j.cell.2013.10.043 (2013).

10 Ye, J. *et al.* TRF2 and Apollo Cooperate with Topoisomerase 2 alpha to Protect Human Telomeres from Replicative Damage. *Cell* **142**, 230-242, doi:10.1016/j.cell.2010.05.032 (2010).

11 Markova, E. *et al.* DNA repair foci and late apoptosis/necrosis in peripheral blood lymphocytes of breast cancer patients undergoing radiotherapy. *International Journal of Radiation Biology* **91**, 934-945, doi:10.3109/09553002.2015.1101498 (2015).

12 Francia, S., Cabrini, M., Matti, V., Oldani, A. & di Fagagna, F. D. DICER, DROSHA and DNA damage response RNAs are necessary for the secondary recruitment of DNA damage response factors. *Journal of Cell Science* **129**, 1468-1476, doi:10.1242/jcs.182188 (2016).

13 Eren, M. K., Kilincli, A. & Eren, O. Resveratrol Induced Premature Senescence Is Associated with DNA Damage Mediated SIRT1 and SIRT2 Down-Regulation. *Plos One* **10**, doi:10.1371/journal.pone.0124837 (2015).
